# National and subnational prevalence and burden of glaucoma in China: A systematic analysis

**DOI:** 10.7189/jogh.07.020705

**Published:** 2017-12

**Authors:** Peige Song, Jiawen Wang, Kajo Bucan, Evropi Theodoratou, Igor Rudan, Kit Yee Chan, on behalf of the Global Health Epidemiology Research Group (GHERG)

**Affiliations:** 1 Centre for Global Health Research, Usher Institute of Population Health Sciences and Informatics, University of Edinburgh, Edinburgh, Scotland, UK; 2 Institute of Medical Humanities, Peking University, Beijing, China; 3 Department of Ophthalmology, University of Split Hospital Centre, Split, Croatia; * Joint last authors

## Abstract

**Background:**

Glaucoma, the second leading cause of blindness, affects approximately 64.3 million individuals worldwide. In China, demographic ageing is in rapid progress. Yet detailed and up–to–date estimates of the scale of glaucoma are rare. We aimed to quantify and understand the prevalence and burden of glaucoma in China from 1990 to 2015, with projections until 2050.

**Methods:**

For this systematic review and meta–analysis, we searched China National Knowledge Infrastructure (CNKI), Wanfang, Chinese Biomedicine Literature Database (CBM–SinoMed), PubMed, Embase and Medline using comprehensive search strategies to identify all relevant articles that have reported the prevalence of glaucoma in the general Chinese population. We used a multilevel mixed–effect meta–regression to estimate the prevalence rates of primary open–angle glaucoma (POAG) and primary angle–closure glaucoma (PACG), and a random–effects meta–analysis to pool the overall prevalence of secondary glaucoma. United Nations population data were used to estimate and project the number of people with glaucoma from 1990 to 2050. Univariable and multivariable meta–regressions were conducted to assess the association between the prevalence of POAG and PACG and relevant demographic and geographic factors. The national burden of POAG and PACG in the years 2000 and 2010 were distributed to six geographic regions accordingly.

**Results:**

From 1990 to 2015, the prevalence of all glaucoma ranged from 2.59% (95% CI = 1.96–3.49) to 2.58% (95% CI = 1.94–3.47). For different subtypes of glaucoma, the overall prevalence of POAG ranged from 1.03% (95% CI = 0.67–1.58) in 1990 to 1.02% (95% CI = 0.67–1.57) in 2015, PACG from 1.41% (95% CI = 1.18–1.68) to 1.40% (95% CI = 1.17–1.68). The overall prevalence of secondary glaucoma was 0.15% (95% CI = 0.10–0.23) during this period. The number of people with all glaucoma in China was 5.92 million (95% CI = 4.47–7.97) in 1990, and 13.12 million (95% CI = 9.88–17.68) in 2015. This increasing trend was also witnessed in different subtypes of glaucoma. The number of people affected by POAG increased from 2.35 million (95% CI = 1.54–3.60) in 1990 to 5.22 million (95% CI = 3.40–7.98) in 2015, PACG from 3.22 million (95% CI = 2.70–3.84) to 7.14 million (95% CI = 5.97–8.53), and secondary glaucoma from 0.34 million (95% CI = 0.23–0.53) to 0.76 million (95% CI = 0.51–1.17). In 2015, more than half (54.42%) of the glaucoma cases were PACG, followed by POAG (39.79%) and secondary glaucoma (5.79%). By 2050, the number of all glaucoma cases in China will be 25.16 million (95% CI = 18.96–33.86). In the multivariable meta–regressions, the odds ratio (OR) for each decade’s increase in age was 1.43 (95% CI = 1.33–1.55) for POAG, and 1.65 (95% CI = 1.51–1.80) for PACG; males were more likely to have POAG (OR 1.36, 95% CI = 1.17–1.59), but less likely to have PACG (OR 0.53, 95% CI = 0.46–0.60) compared with females. After adjustment of age and gender, people living in urban areas were more likely to have POAG compared with those in rural areas (OR 1.54, 95% CI = 1.02–2.35). People in Northeast China were at a higher risk (OR 1.77, 95% CI = 1.07–2.94) of having PACG than people in East China. Among the six regions, East China owed the most POAG and PACG cases, whereas Northwest China owed the least.

**Conclusions:**

This systematic review and meta–analysis suggests a substantial burden of glaucoma in China, with great variances among the different age groups, genders, settings and geographic regions. With the dramatic ageing trend in the next three decades, the prevalence and burden of glaucoma will continue to increase. More elaborate epidemiological studies are needed to optimise public health strategies for mitigating this important health problem.

Glaucoma, the second leading cause of blindness, is an optic neuropathy characterised by progressive structural and functional changes of the optic nerve, leading to a typical appearance of the optic disc and visual field damage if untreated [1–5]. People with glaucoma–induced visual impairment generally suffer from decreased vision–related quality of life (including reduced vision–dependent mobility, increased incidence of falls), and place a huge burden on caregivers and communities [6–9]. Glaucoma is often associated with a long and asymptomatic initial phase, and is usually unnoticed until its later stages, when extensive and irreversible damage has occurred [10,11]. In the late stage of the disease, the effects of medical and surgical treatment can be unsatisfactory, underscoring the importance of early detection and treatment [1,2,12]. As glaucoma has an uncertain prognosis, it requires lifelong management and follow–up to prevent further loss of vision [1,13,14]. The recognition of glaucoma’s pervasive nature and adverse impact on both individuals and society, and the documentation of the magnitude and distribution of glaucoma is of pronounced importance to inform clinicians and researchers, and will guide policymakers in health services allocation [9,15,16].

Globally, 64.3 million individuals, or 3.5% of the world's population, have glaucoma; of these, about 5.7 million people are visually impaired and 3.1 million are blind [5,16]. Of the many subtypes of glaucoma, primary open–angle glaucoma (POAG) is the most common in nearly all regions, accounting for more than two–thirds (68.6%) of all glaucoma cases [15,16]. Geographically, POAG is believed to be particularly prevalent in Africa (4.2%), and least prevalent in Asia (2.3%); however, more than half (53.4%) of the global POAG cases are in Asia due to the relatively large population size of this region [16,17]. Compared with POAG, primary angle–closure glaucoma (PACG) is a less common subtype, but is more visually damaging [18]. PACG also disproportionally affects the global population; it is least common in North America (0.3%), but is the predominant type of glaucoma in Asian populations (1.1%) [16]. More than three quarters (76.7%) of the global PACG cases are in Asia [16,17]. Given the positive association of glaucoma prevalence and advanced age, glaucoma is expected to become an even larger public health concern in the coming decades [18–20]. This dramatic increase of glaucoma burden is also expected to be the case for the largest developing country – China – where rapid ageing of the population is under way [21–23].

The last three decades have seen a proliferation of population–based studies in China. The mounting volume of data on the prevalence of glaucoma in Chinese bibliographical databases allows us to explore the burden of glaucoma in China from a modelling approach [24–26]. However, epidemiological studies on glaucoma to date have been restricted to specific demographic and geographic features in China, and are therefore not generalisable to the overall Chinese population [27–30]. Because of the uncertainty and variation surrounding the epidemiological surveys on glaucoma, a systematic synthesis of the prevalence of glaucoma in China is particularly needed. The first study to pool the prevalence of primary glaucoma in China was published in 2013, which revealed an overall prevalence of 0.7% for POAG, and 1.4% for PACG [30]. The meta–analysis was based on 14 articles from 12 population–based studies published before 2009. No study has yet systematically appraised research into the prevalence of primary glaucoma in China published over the last nine years. Furthermore, considering that different subtypes of glaucoma may require different strategies for screening, prevention and treatment, there is a pressing need for a more updated effort to provide finer quantification of the relative magnitude across the main types of glaucoma, namely, POAG, PACG and secondary glaucoma, which is missing in the 2013 study [17].

To fill the gaps in the evidence matrix, in this study we used a comprehensive systematic review to synthesise the best available evidence from 1990 onwards. Based on the retained evidence, we assessed the prevalence and burden of glaucoma and its subtypes at both the national and subnational levels. The aims of this present study were: 1) to estimate glaucoma prevalence in China by using epidemiological modelling; 2) to estimate and project the overall prevalence and number of people with glaucoma at the national level from 1990 to 2050; and 3) to estimate the regional prevalence and number of people with glaucoma from 2000 to 2010.

## METHODS

### Systematic review

Our comprehensive systematic review was conducted and reported in line with the Preferred Reporting Items for Systematic reviews and Meta–Analyses (PRISMA) guidelines and the Guidelines for Accurate and Transparent Health Estimates Reporting (GATHER) statement [31,32].

#### Search strategy

A systematic literature search was performed to identify all relevant articles that have reported the prevalence of glaucoma in the general Chinese population. The searched databases included three Chinese and three English electronic databases: China National Knowledge Infrastructure (CNKI), Wanfang, Chinese Biomedicine Literature Database (CBM–SinoMed), PubMed, Embase, and Medline. The search strategy combined controlled vocabularies (eg, Medical Subject Heading terms) and free text terms of prevalence (prevalence, incidence, mortality, morbidity, epidemiology), glaucoma and China (China, Chinese, Hong Kong, Macau, Taiwan); the specific search strategies for each database were adapted to fit their specific features (Table S1 in **Online Supplementary Document(Online Supplementary Document)**). We restricted our searches to studies that were published between January 1990 and August 2017. No language restrictions were applied to the searches or search results. The reference lists of all included full–text articles were also scrutinised in detail to identify additional data sources.

#### Inclusion and exclusion criteria

The inclusion and exclusion criteria adopted in this study were developed based on the examination guidelines for glaucoma–related population–based studies [3,16,33]. To be included in this systematic review, studies had to be population–based and report the prevalence of glaucoma. We excluded studies that were hospital–based or conducted in a population that was not representative of the general population. Reviews, commentaries, studies that only adopted qualitative methods and studies that reported the number of eyes with glaucoma instead of the number of individuals were also excluded because they were not able to provide numerical estimates of glaucoma prevalence. Studies that did not include clear assessment methods of glaucoma or relied on self–reported diagnosis were also excluded. Although different case definitions and examination methods exist in identifying glaucoma cases, a remarkable similarity of glaucoma prevalence was noted across surveys despite variations in survey methodology and glaucoma definition [16,34–36]. In this systematic review, we did not exclude studies on the basis of their specific definitions of glaucoma or adopted instrumentation; the assessment of glaucoma should be independent of intraocular pressure (IOP) measurements, but rely on structural or functional evidence of glaucomatous optic neuropathy evaluated by optic disc evaluation or visual field testing [3,16]. Therefore, studies were eligible to contribute data if the following standardised assessments were carried out in suspected cases of glaucoma: anterior chamber angle/depth evaluation by slit–lamp examination or gonioscopy, optic disc evaluation by ophthalmologists using slit–lamp biomicroscopy or fundus photography and visual field testing with automated static perimetry.

#### Study selection and data extraction

Search results from the six bibliographic databases were merged together and duplicate references were removed within and between the databases. All records were independently screened by two researchers (PS and JW) in two stages: screening of titles and abstracts, followed by the retrieval and screening of full–text articles. For multiple articles that reported results of the same individual study, those with the most comprehensive or most recent data were kept. Disagreements were resolved by consensus through discussion.

For the purpose of this study, glaucoma was classified into three main types: POAG, PACG and secondary glaucoma. Relevant data on different subtypes of glaucoma were separately extracted from the studies included. The pilot tested and refined extraction table included three modules:

1. Characteristics of the study: author(s), publication year, study setting (urban, rural or mixed), study location, geographic region, survey year, sampling method, study design (cross–sectional or cohort), whether anterior chamber angle/depth evaluation, IOP measurement, optic disc evaluation and visual field testing were conducted;
2. Characteristics of the investigated population: number of the sample, gender (male, female or mixed), and age (age range, mean or median age, or midpoint of the age range);
3. Prevalence estimates: the number of participants who had been tested and the number of people with glaucoma, by age group, gender, setting and glaucoma subtype, where available.

We classified the sites where the studies were conducted into six geographic regions following definitions of National Bureau of Statistics of China: East China, North China, Northeast China, Northwest China, South Central China, and Southwest China (**Table 1**) [37,38]. Missing data on survey years were imputed for two studies by subtracting three years from the published year, which was based on the average time from survey to publication in studies with available data. In case of censoring age groups, eg, older than 80 years, the same width as other age groups in the same study was used to impute the missing age band.

**Table 1:** The six geographic regions in China

Region	Included provinces
North China	Beijing Municipality, Hebei province, Inner Mongolia Autonomous Region, Shanxi province, Tianjin Municipality;
Northeast China	Heilongjiang province, Jilin province, Liaoning province;
East China	Anhui province, Fujian province, Jiangsu province, Jiangxi province, Shandong province, Shanghai Municipality, Zhejiang province;
South Central China	Guangdong province, Guangxi Zhuang Autonomous Region, Hainan province, Henan province, Hubei province, Hunan province;
Southwest China	Chongqing Municipality, Guizhou province, Sichuan province, Tibet Autonomous Region, Yunnan province;
Northwest China	Gansu province, Ningxia Hui Autonomous Region, Qinghai province, Shaanxi province, Xinjiang Uyghur Autonomous Region;

### Statistical analysis

#### Epidemiological modelling of glaucoma prevalence

Prevalence of POAG, PACG and secondary glaucoma was stabilised by using the logit transformation [39]. In this study, random–effects models were used throughout because of significant heterogeneity in the reported prevalence of POAG, PACG and secondary glaucoma between studies (Table S2 in **Online Supplementary Document(Online Supplementary Document)**). The overall prevalence of secondary glaucoma was derived from the study–specific estimates using a random–effects meta–analysis model (DerSimonian and Laird method) [40]. For POAG and PACG, one individual study might have contributed multiple outcome measurements in the data extraction stage; to take into account the occurrence of different data points from the same study, a multilevel mixed–effect logistic regression approach was adopted [41,42]. Before constructing epidemiological models of the prevalence for POAG and PACG, the association of prevalence estimates and each individual variable, ie, age, gender (male and female), setting (urban, rural and mixed), geographic region, and survey year, was explored using a univariable meta–regression; this was done for POAG and PACG separately. Age and gender were found to be the only common factors that were significantly associated with prevalence estimates of both POAG and PACG. For the purpose of estimating the national prevalence of POAG and PACG, an age– and gender–adjusted model was developed. Given that: 

Then, the prevalence estimates were stabilised by the logit link, 

The prevalence of glaucoma was established as a function of age and gender: 

Therefore, 

And, 

Finally, the age– and gender–specific prevalence of POAG and PACG was generated based on the above–mentioned model. The lower bound of age range was set as 45 years and the upper bound as 89 years because enough data were available for model construction in this broad age range.

#### Estimation of national population with glaucoma from 1990 to 2015

The national number of people with glaucoma (glaucoma “envelopes”) from 1990 to 2015 was derived by multiplying the prevalence of glaucoma with the population in China, available from the United Nations Population Division (UNPD) [43]. For POAG and PACG, the numbers of age– and gender–specific cases were calculated by using their age– and gender–specific prevalence for each 5–year age group estimated in the above models. In view of the limited data availability, the age– and gender–specific prevalence of secondary glaucoma was not estimated, thus the overall prevalence pooled from 12 studies were used (Figure S3 in **Online Supplementary Document(Online Supplementary Document)**). This was performed for the years 1990, 2000, 2010 and 2015 consecutively.

#### Projection of national population with glaucoma from 2020 to 2050

For our projection of people with glaucoma to the year 2050, the prevalence of POAG, PACG and secondary glaucoma was assumed to be constant over the next 33 years. This assumption was partly supported by our multivariable meta–regression model, where no significant changes of POAG and PACG prevalence with survey year were observed after adjusting the effects of age and gender. Bases on the same procedures adopted in the estimation of national population with glaucoma from 1990 to 2015, the numbers of people with glaucoma from 2020 to 2050 were projected by taking UNDP Prospects data, which took into account mortality rates and fertility rates in its population projection [43].

#### Effects of demographic and geographic factors on the prevalence of POAG and PACG

To investigate whether the prevalence of POAG and PACG varied across different subgroups of the population, the associations of prevalence estimates and variables of interest were assessed by multivariable meta–regression, adjusting the effects of age and gender. Before model fitting, all variables were tested for correlation to avoid multicollinearity. The variables were selected based on our knowledge, previous studies and the availability of data in this present study, which included setting (urban, rural and mixed), geographic region, and survey year. As a rule, at least seven data points should be available for each variable [44].

#### Estimation of regional population with POAG and PACG from 2000 to 2010

The regional population with glaucoma was estimated at an envelope condition. This method was initially proposed by the Child Health Epidemiology Reference Group (CHERG) and has been widely adopted in disease burden research [45–47]. The national glaucoma cases were set as the glaucoma envelope, for POAG and PACG separately. Then the “POAG envelope” and the “PACG envelope” were split into the six subnational regions according to the different distributions of risk factors identified in the multivariable meta–regression models. This was conducted for the years 2000 and 2010, where regional population data were available from the fifth and sixth censuses of China [37,38].

All analyses were performed using R, version 3.3.0 (R Foundation for Statistical Computing, Vienna, Austria). The China base map was obtained as a shapefile from the Global Administrative Areas (GADM) database (GADM, 2015, version 2.0; www.gadm.org) and all maps were drawn by ArcMap version 10.1 (Environmental Systems Research Institute, Redlands, CA). A two–sided p–value of less than 0.05 indicated statistically significant difference for all analyses.

## RESULTS

### Summary of systematic review

**Figure 1:**
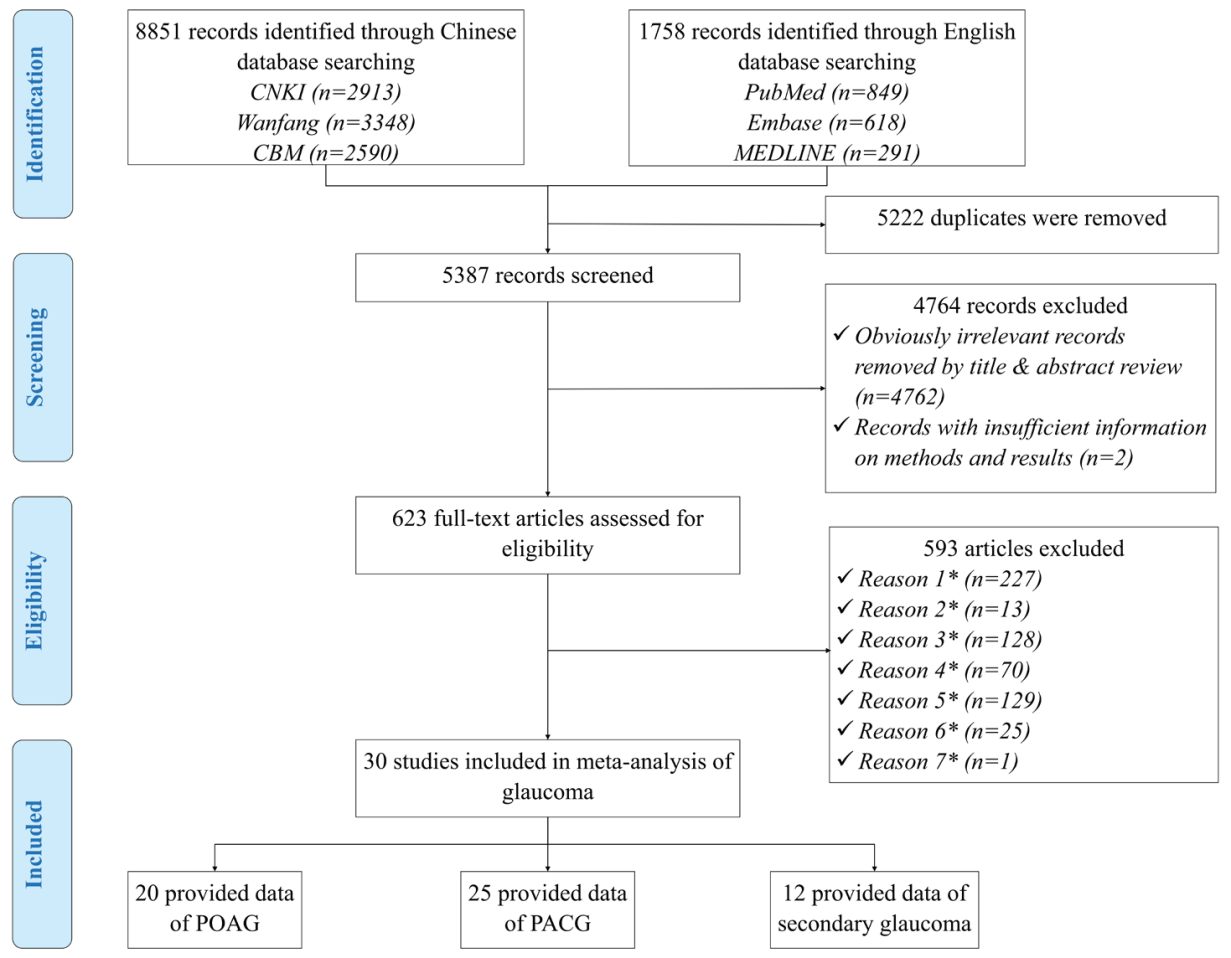
Systematic review flow diagram of studies on glaucoma prevalence in China. Note: *Reason 1–Studies that were not population–based; *Reason 2–Studies that were not based in China; *Reason 3–Papers with no numerical prevalence measure of glaucoma; *Reason 4–Studies that relied on self–reported diagnoses or didn’t conduct standardised assessments (anterior chamber angle/depth evaluation by slit–lamp examination or gonioscopy, optic disc evaluation by ophthalmologists using slit–lamp biomicroscopy or fundus photography and visual field testing with automated static perimetry) in at least glaucoma suspects; *Reason 5–Studies that were conducted in a population with unrepresentative characteristics (diabetic patients, people with reduced vision, etc.); *Reason 6–Multiple publications of the same study; *Reason 7–Papers with inconsistency between reported methods and presented results.

Our initial search identified a total of 10 609 citations for screening, after elimination of duplicates, 5387 records remained. After screening titles and abstracts, 623 potentially relevant full–text articles were reviewed for eligibility, of which 30 reported the prevalence of glaucoma and were included in the systematic review (**Figure 1**). A full list of the included studies is shown in Table S3 in **Online Supplementary Document(Online Supplementary Document)**.

**Figure 2:**
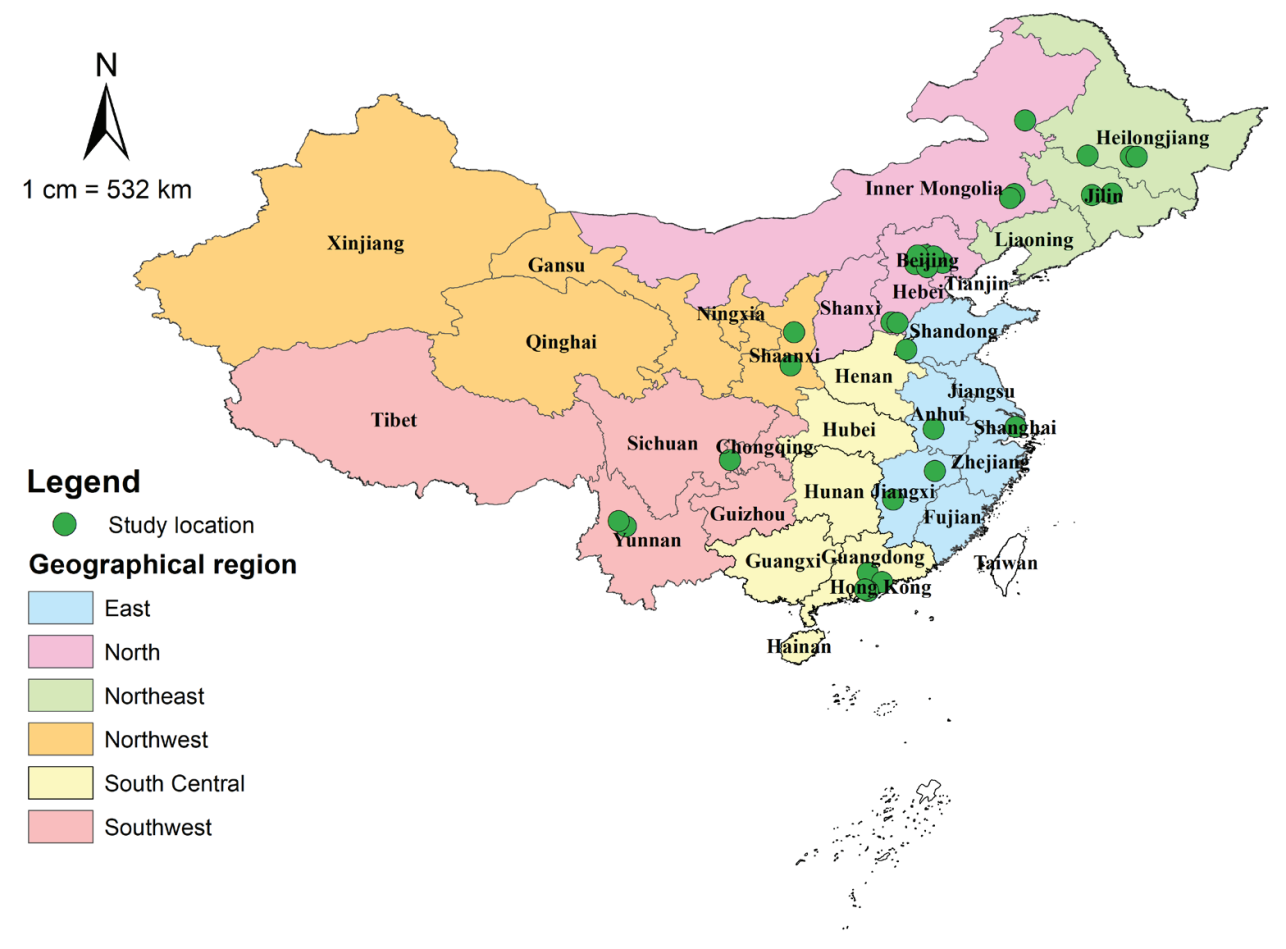
Geographical distribution of the included studies on glaucoma prevalence in China (n = 30).

The 30 studies, published between 1995 and 2016, reported the prevalence of glaucoma with a geographical distribution covering all the six regions in China (**Figure 2**). The included studies were all cross–sectional, of which 20 studies reported the prevalence of POAG, 25 focused on PACG and 12 on secondary glaucoma. The detailed characteristics of every study are listed in Table S4 in **Online Supplementary Document(Online Supplementary Document)**, and the main characteristics of the 30 studies are summarised in **Table 2**. More than half of the 30 studies were published in the past seven years, underlining the necessity for conducting our revision of the estimate. The included studies were generally larger, with the majority (60%, n = 18) being conducted in rural areas. Anterior chamber angle/depth evaluation, IOP measurement and optic disc evaluation were mostly undertaken in all participants, whereas visual field testing was largely used in glaucoma suspects.

**Table 2:** Main characteristics of the included studies on glaucoma prevalence in China (n = 30)

Characteristics of study	Number of studies (%)
**Year published:**	
1990–1999	2 (6.7)
2000–2009	12 (40.0)
2010–2017	16 (53.3)
**Setting:**	
Urban	6 (20.0)
Rural	18 (60.0)
Mixed	6 (20.0)
**Sample size:**	
500–1500	6 (20.0)
1501–2500	7 (23.3)
2501–5000	9 (30.0)
>5000	8 (26.7)
**Geographic regions:**	
North China	12 (40.0)
Northeast China	5 (16.7)
East China	4 (13.3)
South Central China	4 (13.3)
Southwest China	3 (10.0)
Northwest China	2 (6.7)
**Anterior chamber angle/depth evaluation:**	
In all participants	28 (93.3)
In glaucoma suspects	2 (6.7)
**IOP measurement:**	
In all participants	29 (96.7)
In glaucoma suspects	1 (3.3)
**Optic disc evaluation:**	
In all participants	28 (93.3)
In glaucoma suspects	2 (6.7)
**Visual field testing:**	
In all participants	4 (13.3)
In glaucoma suspects	26 (86.7)

### Age– and gender–specific prevalence of POAG and PACG

**Figure 3:**
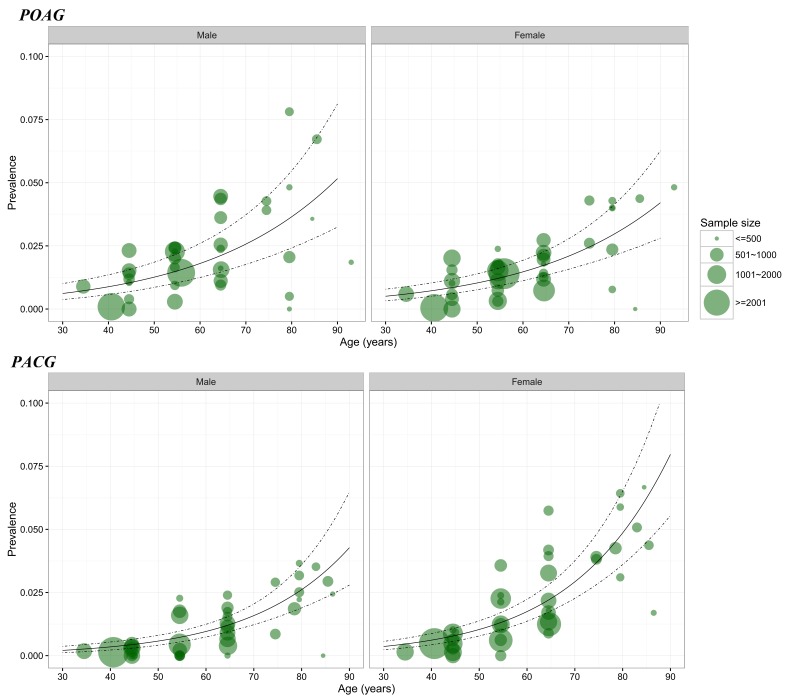
Age– and gender–specific prevalence of primary open–angle glaucoma (POAG) and primary angle–closure glaucoma (PACG) based on the informative data points from the included studies. Note: The size of each bubble is proportional to the sample size. Overall, there were 86 data points for constructing the gender–specific relation between age and prevalence for POAG, and 103 for PACG.

Based on a substantial number of data points from the included studies, the gender–specific relationship between age and the prevalence of POAG and PACG was constructed (**Figure 3**). The informative data points covered a wide age spectrum from the mid–30s to the 9th decade, with the majority concentrating between the mid–40s to the mid–80s. Therefore, in the estimation of age– and gender–specific prevalence of POAG and PACG, the age range was set as from 45 years to 89 years.

**Figure 4:**
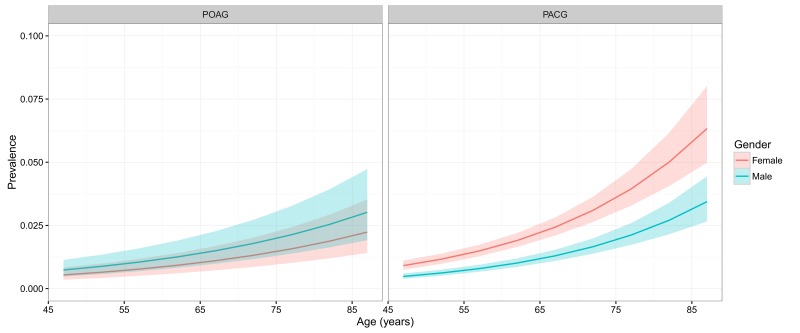
Estimated age– and gender–specific prevalence of primary open–angle glaucoma (POAG) and primary angle–closure glaucoma (PACG) in China, with 95% confidence intervals.

**Table 3** and **Figure 4** show the estimated age– and gender–specific prevalence of POAG and PACG respectively. Generally, the prevalence of POAG and PACG both increased steadily with advanced age, and this positive relationship between age and prevalence rate was similar between genders, but more pronounced for PACG. In males, the prevalence of POAG ranged from 0.74% (95% CI = 0.48–1.14) in individuals aged 45–49 years to 3.02% (95% CI = 1.92–4.73) in those aged 85–89 years. The prevalence of POAG in females was slightly lower than that in males across the whole age spectrum from 45 to 89 years, ranging from 0.54% (95% CI = 0.35–0.84) to 2.24% (95% CI = 1.41–3.53). In contrast, the prevalence of PACG was consistently higher in females than in males. In females, the prevalence of PACG ranged from 0.91% (95% CI = 0.74–1.11) in those aged 45–49 years to 6.33% (95% CI = 4.98–8.02) in those aged 85–89 years. In males, the prevalence of PACG increased from 0.48% (95% CI = 0.39–0.60) in people aged 45–49 years to 3.44% (95% CI = 2.66–4.45) in elderly aged 85–89 years.

**Table 3:** Estimated gender–specific prevalence of POAG and PACG in China, by age group

Age (years)	Prevalence of POAG (%, 95% CI)		Prevalence of PACG (%, 95% CI)	
**Male**	**Female**	**Male**	**Female**
45–49	0.74	0.54	0.48	0.91
	(0.48–1.14)	(0.35–0.84)	(0.39–0.60)	(0.74–1.11)
50–54	0.88	0.65	0.62	1.16
	(0.57–1.34)	(0.42–0.99)	(0.51–0.75)	(0.98–1.38)
55–59	1.05	0.77	0.79	1.49
	(0.69–1.60)	(0.51–1.18)	(0.66–0.94)	(1.28–1.74)
60–64	1.25	0.92	1.01	1.90
	(0.83–1.90)	(0.61–1.41)	(0.86–1.20)	(1.65–2.20)
65–69	1.50	1.10	1.30	2.43
	(0.98–2.27)	(0.72–1.68)	(1.09–1.54)	(2.10–2.81)
70–74	1.79	1.32	1.66	3.10
	(1.17–2.72)	(0.86–2.02)	(1.38–1.99)	(2.64–3.64)
75–79	2.13	1.57	2.12	3.94
	(1.38–3.27)	(1.02–2.43)	(1.73–2.59)	(3.28–4.73)
80–84	2.54	1.87	2.70	5.00
	(1.63–3.93)	(1.20–2.92)	(2.15–3.39)	(4.06–6.16)
85–89	3.02	2.24	3.44	6.33
	(1.92–4.73)	(1.41–3.53)	(2.66–4.45)	(4.98–8.02)

POAG – primary open–angle glaucoma, PACG – primary angle–closure glaucoma, CI – confidence interval

### National population with glaucoma from 1990 to 2015

By extrapolating the estimated age– and gender–specific prevalence of POAG and PACG to UNPD data, the numbers of people with POAG and PACG were generated (Table S5 in **Online Supplementary Document(Online Supplementary Document)**). At the national level, the overall prevalence of glaucoma was listed in **Table 4**. From 1990 to 2015, the prevalence of all glaucoma ranged from 2.59% (95% CI = 1.96–3.49) to 2.58% (95% CI = 1.94–3.47), indicating a slightly relative decreasing rate of 0.39%. For different subtypes of glaucoma, the overall prevalence of POAG ranged from 1.03% (95% CI = 0.67–1.58) in 1990 to 1.02% (95% CI = 0.67–1.57) in 2015, which yielded a relative decreasing rate of 0.97%. Similarly, the prevalence of PACG decreased by 0.71%, from 1.41% (95% CI = 1.18–1.68) in 1990 to 1.40% (95% CI = 1.17–1.68) in 2015. Based on 12 individual studies, the overall prevalence of secondary glaucoma was pooled as 0.15% (95% CI = 0.10–0.23) and assumed as constant over the time frame of this research (Figure S3 in **Online Supplementary Document(Online Supplementary Document)**).

**Table 4:** Estimated prevalence and number of people with glaucoma in China from 1990 to 2015, by glaucoma type

Glaucoma type	Prevalence of glaucoma (%, 95% CI)		Number of people with glaucoma (million, 95% CI)		Relative rate of change (%, 1990–2015)	
**1990**	**2015**	**1990**	**2015**	**Prevalence**	**Cases**
POAG	1.03	1.02	2.35	5.22	–0.97	122.13
	(0.67–1.58)	(0.67–1.57)	(1.54–3.60)	(3.40–7.98)		
PACG	1.41	1.40	3.22	7.14	–0.71	121.74
	(1.18–1.68)	(1.17–1.68)	(2.70–3.84)	(5.97–8.53)		
Secondary glaucoma	0.15		0.34	0.76	–	123.53
	(0.10–0.23)		(0.23–0.53)	(0.51–1.17)		
All glaucoma*	2.59	2.58	5.92	13.12	–0.39	121.62
	(1.96–3.49)	(1.94–3.47)	(4.47–7.97)	(9.88–17.68)		

POAG – primary open–angle glaucoma, PACG – primary angle–closure glaucoma, CI – confidence interval

*All glaucoma includes POAG, PACG and secondary glaucoma.

**Figure 5:**
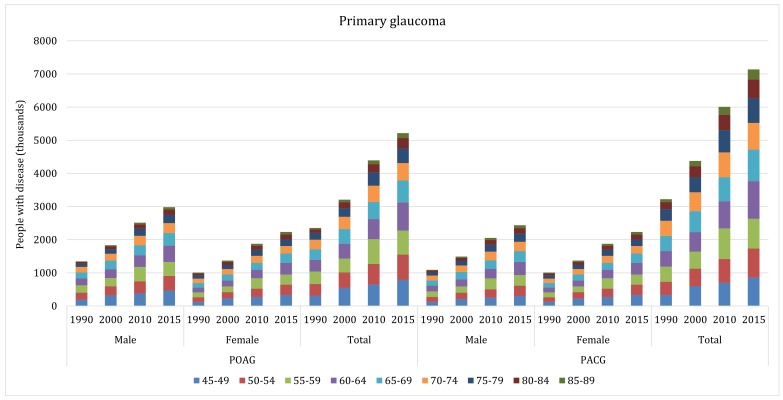
Estimated gender–specific number of people with primary open–angle glaucoma (POAG) and primary angle–closure glaucoma (PACG) in China from 1990 to 2015, with contributing age groups.

With the ageing of Chinese population during 1990–2015, the total number of people living with glaucoma increased dramatically (**Table 4**). The number of people with all glaucoma in China was 5.92 million (95% CI = 4.47–7.97) in 1990 and 13.12 million (95% CI = 9.88–17.68) in 2015, indicating an overall increasing rate of 121.62% throughout this period. This increasing trend was also witnessed in different subtypes of glaucoma. For POAG, the affected cases increased by 122.13%, from 2.35 million (95% CI = 1.54–3.60) in 1990 to 5.22 million (95% CI = 3.40–7.98) in 2015. Similarly, the number of people with PACG increased by 121.74%, ranging from 3.22 million (95% CI = 2.70–3.84) in 1990 to 7.14 million (95% CI = 5.97–8.53) in 2015. Even for secondary glaucoma, whose overall prevalence was assumed as constant, the number of affected people also increased by 123.53% during 1990 to 2015, from 0.34 million (95% CI = 0.23–0.53) to 0.76 million (95% CI = 0.51–1.17). In 2015, more than half (54.42%) of the glaucoma cases were PACG, followed by POAG (39.79%) and secondary glaucoma (5.79%). From 1990 to 2015, the age group that contributed the most cases shifted from 55–59 years to 60–64 years for both POAG and PACG (**Figure 5**).

### Projection of national population with glaucoma from 2020 to 2050

In our projection analysis, the age– and gender–specific prevalence estimates of POAG and PACG, and the overall prevalence estimate of secondary glaucoma were all assumed as constant. By applying these estimates to the UNPD data up to the year 2050, the numbers of people with glaucoma were projected (Table S5 in **Online Supplementary Document(Online Supplementary Document)** and **Table 5**). Unlike the slight declining trend of glaucoma prevalence between 1990 and 2015, from 2020 to 2050, the overall prevalence of all glaucoma is expected to increase from 2.64% (95% CI = 1.99–3.55) to 3.48% (95% CI = 2.63–4.69), which is a 32% increase. For different subtypes of glaucoma, the prevalence of POAG will also increase during this period, but at a lower rate (27%). In 2020, the prevalence of POAG is projected to be 1.05% (95% CI = 0.68–1.60), and then reach 1.33% (95% CI = 0.86–2.04) in 2050. The prevalence of PACG will show a greater increasing rate between 2020 and 2050, from 1.44% (95% CI = 1.21–1.72) to 2.01% (95% CI = 1.66–2.42), ie, by 40%.

**Table 5:** Projected prevalence and number of people with glaucoma in China from 2020 to 2050, by glaucoma type

Glaucoma type	Prevalence of glaucoma (%, 95% CI)		Number of people with glaucoma (million, 95% CI)		Relative rate of change (%, 2020–2050)	
**2020**	**2050**	**2020**	**2050**	**Prevalence**	**Cases**
POAG	1.05	1.33	6.06	9.59	26.67	58.25
	(0.68–1.60)	(0.86–2.04)	(3.95–9.27)	(6.23–14.72)		
PACG	1.44	2.01	8.36	14.49	39.58	73.33
	(1.21–1.72)	(1.66–2.42)	(7.00–9.98)	(12.01–17.48)		
Secondary glaucoma	0.15		0.87	1.08	–	24.14
	(0.10–0.23)		(0.58–1.33)	(0.72–1.66)		
All glaucoma*	2.64	3.48	15.28	25.16	31.82	64.66
	(1.99–3.55)	(2.63–4.69)	(11.53–20.58)	(18.96–33.86)		

POAG – primary open–angle glaucoma, PACG – primary angle–closure glaucoma, CI – confidence interval

*All glaucoma includes both primary glaucoma and secondary glaucoma.

**Figure 6:**
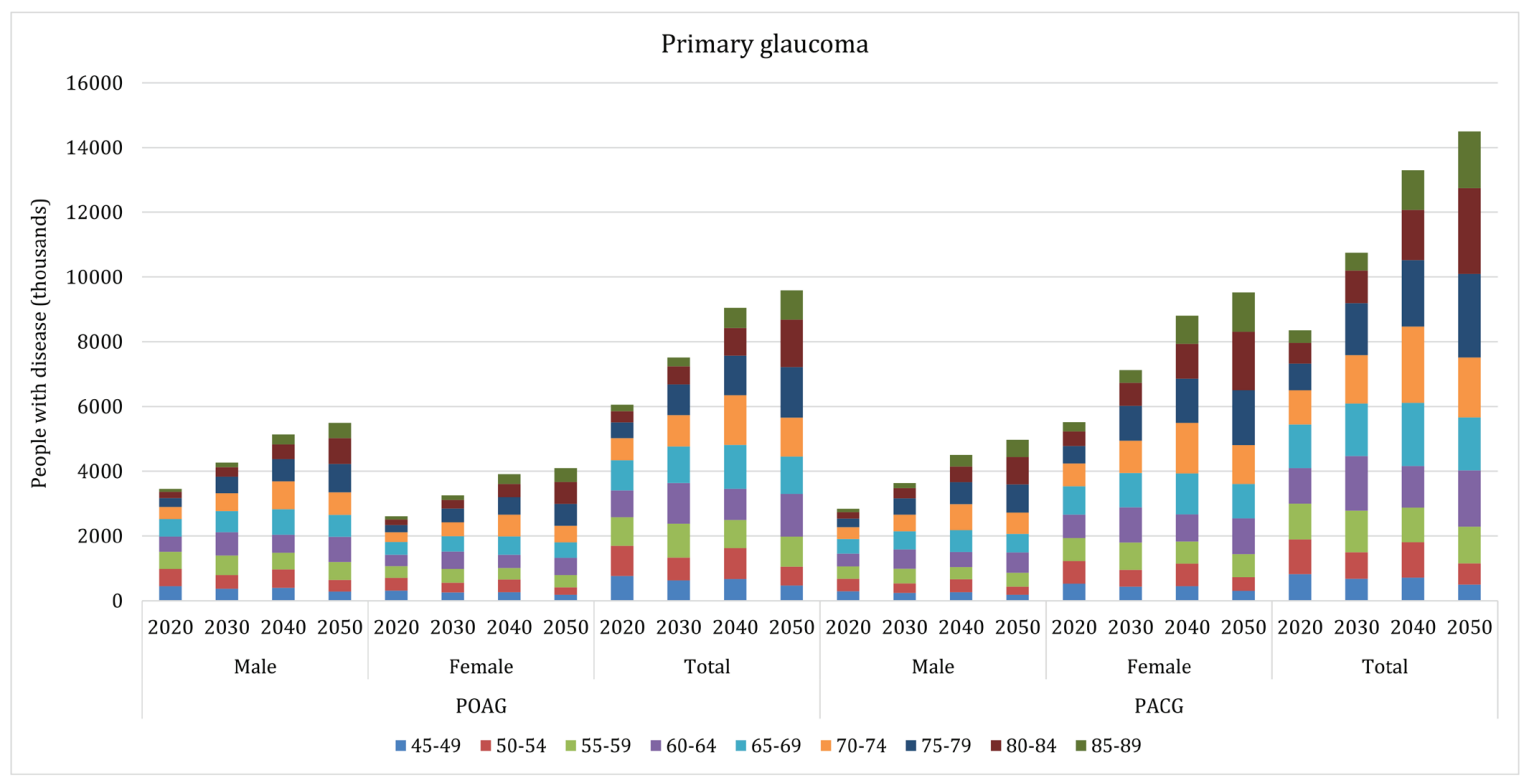
Projected gender–specific number of people with primary open–angle glaucoma (POAG) and primary angle–closure glaucoma (PACG) in China from 2020 to 2050, with contributing age groups.

The projected number of people living with glaucoma in China is also shown in **Table 5**. Between 2020 and 2050, the number of all glaucoma cases in China is expected to increase from 15.28 million (95% CI = 11.53–20.58) to 25.16 million (95% CI = 18.96–33.86), ie, by 65%. The increasing rates for POAG and PACG will also be notable within the same period. The number of people with POAG is expected to increase from 6.06 million (95% CI = 3.95–9.27) to 9.59 million (95% CI = 6.23–14.72), and those with PACG from 8.36 million (95% CI = 7.00–9.98) to 14.49 million (95% CI = 12.01–17.48), which will translate into the rates of increase between 2020 and 2050 of 58% and 73%, respectively. Due to the forecasted trend in population ageing over the next three decades, the number of secondary glaucoma cases is anticipated to also increase slightly, from 0.87 million (95% CI = 0.58–1.33) in 2020 to 1.08 million (95% CI = 0.72–1.66) in 2050; ie, an increase by 24.14%. During this period, PACG will remain the predominant subtype of glaucoma in China, followed by POAG and secondary glaucoma. From 2020 to 2050, the age group in which most POAG cases will be concentrated will shift from 65–69 years to 75–79 years, and the age group for PACG will shift from 65–69 years to 80–84 years (**Figure 6**).

### Effects of demographic and geographic factors on the prevalence of POAG and PACG

Based on univariable and multivariable meta–regression models (**Table 6**), the effects of selected demographic and geographic factors on the risk of POAG and PACG were assessed. The univariable meta–regression indicated that age, gender and study setting were significantly associated with POAG, while PACG was associated with age, gender and geographic region. No evidence for secular trends was observed. After adjusting for age and gender in a multivariable meta–regression, the odds ratio (OR) for each increase in age by 10 years was 1.43 (95% CI = 1.33–1.55) for POAG, and 1.65 (95% CI = 1.51–1.80) for PACG. Males still showed a higher risk of POAG (1.36, 95% CI = 1.17–1.59), but a lower risk of PACG (0.53, 95% CI = 0.46–0.60) in comparison with females. People living in urban areas were more likely to have POAG compared to those in rural areas, with an OR of 1.54 (95% CI = 1.02–2.35). Among the six geographic regions, people in Northeast China were at a higher risk (1.77, 95% CI = 1.07–2.94) of having PACG than people in East China.

**Table 6:** Odds ratios for POAG and PACG in terms of age, gender, setting and geographic region from multilevel univariable and multivariable meta–regression models, with 95% confidence intervals

Variable	Unadjusted		Age and gender–adjusted	
**POAG**	**PACG**	**POAG**	**PACG**
Age (per decade)	1.38 (1.30–1.47)*	1.58 (1.49–1.67)*	1.43 (1.33–1.55)*	1.65 (1.51–1.80)*
**Gender:**†				
Female	Reference	Reference	Reference	Reference
Male	1.39 (1.19–1.62)*	0.53 (0.46–0.60)*	1.36 (1.17–1.59)*	0.53 (0.46–0.60)*
**Setting:**				
Rural	Reference	Reference	Reference	Reference
Urban	1.68 (1.13–2.51)*	0.90 (0.64–1.28)	1.54 (1.02–2.35)*	0.82 (0.54–1.23)
Mixed	0.93 (0.37–2.33)	1.13 (0.67–1.90)	2.18 (0.68–6.99)	0.64 (0.25–1.62)
**Geographic region:**				
East	Reference	Reference	Reference	Reference
North	1.44 (0.45–4.61)	1.81 (0.93–3.52)	1.29 (0.36–4.69)	1.23 (0.77–1.97)
Northeast	1.48 (0.31–7.02)	2.87 (1.37–6.01)*	1.41 (0.26–7.74)	1.77 (1.07–2.94)*
Northwest	0.80 (0.10–6.33)	1.87 (0.79–4.41)	0.44 (0.05–4.07)	1.38 (0.76–2.49)
South Central	2.14 (0.45–10.14)	1.54 (0.72–3.28)	–	0.89 (0.51–1.53)
Southwest	1.48 (0.37–6.00)	1.51 (0.69–3.29)	0.94 (0.17–5.16)	1.24 (0.70–2.22)
**Investigation year (per decade)**‡	1.07 (0.58–1.98)	0.76 (0.50–1.15)	1.47 (0.51–4.25)	1.18 (0.92–1.50)

POAG – primary open–angle glaucoma, PACG – primary angle–closure glaucoma, CI – confidence interval

*Statistically significant.

†The effect of gender was estimated based on studies that reported gender–specific glaucoma prevalence.

‡The secular trend was evaluated based on studies that were conducted after the year 2000.

### Regional population with POAG and PACG from 2000 to 2010

By taking the effects of age, gender and setting, the national POAG cases were distributed to the six geographic regions in China (**Table 7**). In 2000, the overall prevalence of POAG was 1.01% (95% CI = 0.66–1.55) in China, ranging from 0.96% (95% CI = 0.60–1.53) in Southwest China to 1.08% (95% CI = 0.75–1.54) in Northeast China. In 2010, the overall prevalence of POAG in China rose to 1.03% (95% CI = 0.67–1.57), and the regions with the highest prevalence of POAG were Northeast China (1.05%, 95% CI = 0.71–1.54) and East China (1.05%, 95% CI = 0.69–1.59), and that with the lowest POAG prevalence was Northwest China (0.98%, 95% CI = 0.62–1.53). From 2000 to 2010, the prevalence of POAG has risen in China, with an exception of Northeast China, where the prevalence of POAG decreased by 2.47%. The most marked increasing rate was observed in Southwest China (3.68%). In both 2000 and 2010, the distribution of POAG cases across the six geographic regions was similar, with the most cases in East China and the least in Northwest China. From 2000 to 2010, the greatest increase rate was in North China (46.13%), and the least in Southwest China (26.51%).

**Table 7:** Estimated prevalence and number of people with POAG in China from 2000 to 2010, by geographic region

Region	Prevalence of POAG (%, 95% CI)		Number of people with POAG (million, 95% CI)		Relative rate of change (%, 2000–2010)	
**2000**	**2010**	**2000**	**2010**	**Prevalence**	**Cases**
North China	1.01	1.02	0.38	0.55	1.00	46.13
	(0.67–1.53)	(0.68–1.54)	(0.25–0.57)	(0.37–0.83)		
Northeast China	1.08	1.05	0.30	0.44	–2.47	45.03
	(0.75–1.54)	(0.71–1.54)	(0.21–0.43)	(0.30–0.64)		
East China	1.04	1.05	1.02	1.39	1.00	36.34
	(0.69–1.58)	(0.69–1.59)	(0.67–1.54)	(0.91–2.10)		
South Central China	1.01	1.01	0.83	1.13	0.72	35.65
	(0.65–1.56)	(0.65–1.57)	(0.53–1.29)	(0.73–1.75)		
Southwest China	0.96	1.00	0.48	0.61	3.68	26.51
	(0.60–1.53)	(0.63–1.59)	(0.30–0.77)	(0.38–0.97)		
Northwest China	0.97	0.98	0.19	0.27	1.00	41.49
	(0.62–1.51)	(0.62–1.53)	(0.12–0.30)	(0.18–0.43)		
China	1.01	1.03	3.21	4.39	1.05	36.97
	(0.66–1.55)	(0.67–1.57)	(2.09–4.91)	(2.86–6.72)		

POAG – primary open–angle glaucoma, PACG – primary angle–closure glaucoma, CI – confidence interval

The number of people with PACG in China was distributed based on the multivariable meta–regression model of PACG and features of the six geographic regions (**Table 8**). In 2000, the overall prevalence of PACG in China was 1.38% (95% CI = 1.16–1.65), with the highest regional prevalence estimate in Northeast China (2.04%, 95% CI = 1.82–2.26) and lowest in South Central China (1.12%, 95% CI = 0.95–1.32). In 2010, the national prevalence of PACG rose by 1.30%, reaching to 1.40% (95% CI = 1.17–1.68), and the region with the highest prevalence of PACG was still Northeast China (2.06%, 95% CI = 1.84–2.30) and that with the lowest PACG prevalence was still South Central China (1.11%, 95% CI = 0.94–1.31). Within this time frame, the prevalence of PACG decreased in North China, East China and South Central China, but increased in Northeast China, Southwest China and Northwest China. The region with the greatest increasing rate of PACG prevalence was Southwest China (5.06%), and that with the greatest decreasing rate was South Central China (–0.52%). From 2000 to 2010, East China consistently harboured the largest share of PACG cases, while Northwest China has had the smallest share. Overall, the number of people with PACG increased from 4.37 million (95% CI = 3.66–5.23) to 6.01 million (95% CI = 5.03–7.18), which is a 37% increase over this period. This increase was also witnessed in every region, being the most marked in Northeast China (51%) and the least in Southwest China (28%).

**Table 8:** Estimated prevalence and number of people with PACG in China from 2000 to 2010, by geographic region

Region	Prevalence of PACG (%, 95% CI)		Number of people with PACG (million, 95% CI)		Relative rate of change (%, 2000–2010)	
**2000**	**2010**	**2000**	**2010**	**Prevalence**	**Cases**
North China	1.48	1.47	0.55	0.79	–0.47	44.00
	(1.42–1.53)	(1.41–1.52)	(0.53–0.57)	(0.76–0.82)		
Northeast China	2.04	2.06	0.57	0.86	1.27	50.59
	(1.82–2.26)	(1.84–2.30)	(0.51–0.64)	(0.77–0.96)		
East China	1.27	1.27	1.24	1.68	–0.03	34.94
	(0.99–1.63)	(0.99–1.63)	(0.97–1.59)	(1.30–2.15)		
South Central China	1.12	1.11	0.93	1.24	–0.52	33.99
	(0.95–1.32)	(0.94–1.31)	(0.79–1.09)	(1.05–1.46)		
Southwest China	1.51	1.59	0.76	0.97	5.06	28.20
	(1.22–1.87)	(1.28–1.97)	(0.61–0.94)	(0.78–1.20)		
Northwest China	1.60	1.63	0.32	0.46	1.58	42.31
	(1.26–2.03)	(1.27–2.07)	(0.25–0.41)	(0.36–0.58)		
China	1.38	1.40	4.37	6.01	1.30	37.31
	(1.16–1.65)	(1.17–1.68)	(3.66–5.23)	(5.03–7.18)		

POAG – primary open–angle glaucoma, PACG – primary angle–closure glaucoma, CI – confidence interval

## DISCUSSION

Based on rigorous systematic review of existing evidence on glaucoma prevalence in China, this study offers a comprehensive estimate of the prevalence and burden of glaucoma in China at both national and subnational levels, and compares the relative magnitude of three main subtypes of glaucoma, ie, POAG, PACG and secondary glaucoma, in the general mainland Chinese population. From 1990 to 2015, the prevalence of glaucoma fluctuated at around 2.6%, corresponding to 5.92 million and 13.12 million people with glaucoma in the years 1990 and 2015, respectively. By 2050, the prevalence of glaucoma will rise to 3.48%, which equivalents to a total of 25.16 million affected people. Substantial evidence demonstrated that PACG was the predominant subtype of glaucoma in the general Chinese population, followed by POAG and secondary glaucoma. The geographic variations in the prevalence of POAG and PACG were also assessed, with urban dwellers at a higher risk of developing POAG than rural dwellers, and people living in Northeast China being more prone to PACG than people in East China. Because of the uneven population distribution in China, from 2000 to 2010, East China consistently had the largest share of both POAG and PACG cases, and Northwest China the least.

To the best of our knowledge, this study is the most up–to–date and comprehensive systematic review and meta–analysis to explore and present the national and subnational prevalence and burden of glaucoma in China. The principal strengths of this study are a reasonable coverage of the Chinese population, a comprehensive literature search, and a stringent approach to selecting studies for inclusion. Ultimately, this systematic review was built upon 30 individuals studies, which was more than double the number of studies included in the first systematic review on glaucoma in China [30]. With a wide geographical scope covering all the six geographic regions of China, the included studies ensured a sufficient power to conduct the estimates for both the whole nation and subnational regions. Furthermore, with the aim of limiting between–study heterogeneity due to methodological variations, the assessments of glaucoma in the studies included were based on structural or functional evidence of glaucomatous optic neuropathy, rather than IOP measurements, which in part guaranteed a very good detection ability of early–stage glaucoma. In addition, POAG in this study included persons with IOP at all levels [3,16]. Moreover, the prevalence and burden of secondary glaucoma in China was developed for the first time, which added new evidence to the epidemiology of glaucoma both domestically and globally.

Despite the strengths of this study, there are also multiple limitations. First, given the diversity of studies included in study design, targeted population, methods and settings, a relatively high degree of heterogeneity among studies included was observed. Although the estimates of POAG and PACG prevalence were generated based on meta–regression, by taking the effects of age, gender and geographic factors together, some factors other than chance may also be attributable to the observed variance, but could not be fully controlled. In this study, a key issue was that we didn’t choose to exclude studies based on consensus criteria for the definitions and grading systems of glaucoma, but rather relied on the examinations. This is because previous studies suggested a remarkable similarity among surveys with different survey methods and glaucoma definitions [16,34–36]. This approach for defining eligible studies has been widely adopted in previous systematic reviews on glaucoma prevalence, but it might still be influenced by the inherent subjectivity of interpreting ophthalmic images [16,17,34,35]. Second, compared with primary glaucoma, secondary glaucoma has been under–examined in epidemiological studies [16,17,48]. In this study, despite our extensive efforts to identify all the available evidence without language restrictions, the number of eligible studies that provided the estimate of secondary glaucoma prevalence was still not sufficient. Given that there was moderate heterogeneity between studies that reported the prevalence of secondary glaucoma, we acknowledge issues about the appropriateness of roughly reporting an overall prevalence of secondary glaucoma. Third, the projections of glaucoma were only based on the assumption that the prevalence estimates will be constant, thus, changes in the number of people with glaucoma only reflect changes in demographic features of the next three decades. Although this assumption has been commonly adopted in the projection of disease burden, the power of projection analysis beyond the period of studies conducted is limited [16,17,49]. Forth, only the effects of age, gender, setting and geographic region were assessed in subgroup analyses by using both univariable and multivariable meta–regression; however, other relevant factors that were not obtained from the included studies may have also had a role. In addition, all these factors were aggregate level data, thus hampering the opportunity to explore the differences in effects at the individual level, or interaction between factors[50,51]. Fifth, the estimates of glaucoma prevalence were generated at the regional level at best. Any estimates at the provincial level were not possible, owing to the limited availability of data in each province. Taken collectively, the results presented in this study should be interpreted with caution.

The overall estimated prevalence of POAG in this study was slightly higher than that in the previous systematic review (1.0% vs 0.7%) [30]. The disparity between these two estimates might be explained by the combined effect of the different age and gender structures, and the different geographic features of the participants included in these two systematic reviews. Surprisingly, despite the substantial variation in the studies that were included as the basis for both reports, and further differences in adopted methods of meta–analysis, the prevalence of PACG in these two studies was almost identical – both at the level of 1.4%. This similarity in PACG prevalence supports the current understanding of the magnitude of PACG in China.

The prevalence estimates of POAG and PACG were notably associated with advanced age in both sexes; this strong positive relationship matches the natural history of primary glaucoma, which was described in many previous studies. This association confirms the commonly accepted notion that primary glaucoma is an age–related disease [36,52–56]. With increasing longevity, a striking increase in the prevalence and burden of glaucoma is likely, especially for primary glaucoma.

The distribution patterns of POAG and PACG by gender were opposite, with POAG being the predominant subtype of glaucoma in males, and PACG in females. The female predilection for PACG has been widely acknowledged in previous studies, and could be linked to the aetiology of disease, differences in biological factors and environmental exposures between sexes [30,55–58]. However, the evidence of gender effect on POAG is still conflicting [16,34,35,57]. The findings in the present study disagree with the first systematic review of glaucoma prevalence in China, where male predilection for POAG was not reported [30]. The discrepancy between these two studies might be explained by the inadequate study power to confirm associations. Further studies are still needed to explore the different effects of gender on the development of glaucoma, especially POAG, and for deciding different public health policies.

In view of the general understanding that secondary glaucoma is caused by other ocular or systematic disorders that may lead to an increase in intraocular pressure, rather than a normal degenerative process with ageing, it was expected that no effects of age, gender or geographic factors would be seen [48,53]. Only a pooled overall prevalence was generated for secondary glaucoma, with no separate subgroup analysis. In this study, the prevalence of secondary glaucoma was largely lower than that for East Asia (0.15% vs 0.39%) [17]. However, this relatively lower prevalence of secondary glaucoma, presented in this study, still needs to be confirmed with new data. There is little doubt that secondary glaucoma is less frequent in comparison with primary glaucoma; however, the disease burden caused by secondary glaucoma should never be underestimated or neglected, bearing in mind its visually destructive effects [53,54].

In China, PACG was estimated to be responsible for the largest share of glaucoma, followed by POAG and secondary glaucoma. This finding confirms previous studies, which concluded that Chinese people might be more likely to develop PACG than any other ethnic groups in the world [36,59]. Mechanisms underlying this phenomenon are controversial, but may be associated with the difference of anterior chamber and angle anatomy among races [18,60,61]. In addition, given that PACG is more common in females than males, and females have a longer life expectancy than males, the burden of PACG is considerably concerning [43]. The visual damages of PACG are more severe than of the other main subtypes of glaucoma, presenting an even greater public health challenge with a considerable social and economic impact [18,21].

The higher prevalence of POAG in urban than in rural settings is in agreement with findings from studies conducted in China, and also with many other regions across the world [16,17,30]. The reasons for this are not certain, but may be partly related to a higher myopia prevalence in urban areas, and to other potential risk factors for POAG that vary greatly between urban and rural areas. Hypertension, diabetes, diet, physical activity and air pollution may also play a role [16,17,62,63]. With rapid urbanisation, the prevalence and burden of POAG may continue to increase in China [64,65]. For PACG, people in Northeast China had the highest prevalence. An explanation for this geographic variation is an evolutionary modification of shallower anterior chambers that resist corneal freezing [66,67]. However, these geographic variations might also be a product of differences in the studies included among regions. Future studies should be undertaken to assess geographical risk factors for glaucoma in more detail, to improve locally relevant policy–making on glaucoma.

The findings of this study add insight to our knowledge of the epidemiology of glaucoma and have clear policy implications for China. Together with an ageing demography, glaucoma, especially primary glaucoma, will place an ever–increasing burden on the already stretched health care services in China, unless proactive preventive strategies are put in place. Despite advances in medical treatment, a cost–effective approach for detecting and diagnosing glaucoma is still lacking [68–71]. Indeed, a strong need remains for the development of an appropriate prevention and treatment framework to counter the growing burden of glaucoma in China, especially in rural and poor areas where medical resources are unevenly distributed. National and local efforts are also needed for the formulation of better medical systems and effective public health strategies informed by evidence, such as reallocating medical resources, improving access to health care, and health education on the importance of early examination.

In the meantime, the need to scale up reliable data on glaucoma epidemiology in places where primary data have never been available has been highlighted in the present study. This is essential for both researchers and policymakers to improve understanding of the magnitude and distribution of this problem and the main risk factors. For a comprehensive assessment of glaucoma epidemiology in China, more robust evidence from studies using consistent methods across populations and further reviews of the prevalence of glaucoma is needed to corroborate the statements in the present study more reliably.

In conclusion, this contemporary systematic review and meta–analysis suggests a substantial burden of glaucoma in China, with considerable variation among the different age groups, genders, study settings and geographic regions. PACG is the predominant subtype of glaucoma in the general Chinese population, followed by POAG and secondary glaucoma. In the next three decades, the prevalence and burden of glaucoma will continue to increase with the current ageing trend. More elaborate epidemiological studies are needed to optimise public health strategies for mitigating this important health problem.
