# Supplementary material for: National and subnational prevalence and burden of glaucoma in China: A systematic analysis
Source: J Glob Health. 2017 Dec 22;7(2):020705. doi: 10.7189/jogh.07.020705 (PMC5737099; doi:10.7189/jogh.07.020705)
Supplement: Online Supplementary Document [file jogh-07-020705-s001.pdf]

Online Supplementary Document

Song et al. National and subnational prevalence and burden of glaucoma in China: A systematic analysis

J Glob Health 2017;7:020705

Table S1. Search strategy to identify studies reporting the prevalence of glaucoma in China

CNKI
Access Date: 18 Aug 2017
Subject category: Medicine & Public Health
Sub-database: Journal, Featured journal, Doctoral dissertation, Master dissertation
检索表达式：
(SU % '青光眼') AND (SU % '发病率' + '发生率' + '患病率'+ '罹患率' + '现患率'+ '死亡率' + '病死率'+ '流行' + '负担'+ '现况调查'+ '现况研究')
发表时间：从 1990-01-01 到 2017-08-18
Search Terms: (SU % 'qingguangyan') AND (SU % 'fabinglv' + 'fashenglv' + 'huanbinglv'+ 'lihuanlv' + 'xianhuanlv'+ 'siwanglv' + 'bingsilv'+ 'liuxing' + 'fudan'+ 'xiankuangdiaocha'+ 'xiankuangyanjiu')
Published time：From 01/01/1990 to 26/06/2016

Wanfang
Access Date: 18 Aug 2017
Sub-database: Journal articles, Dissertations
检索表达式：检索表达式：(主题:(青光眼)) * (主题:(发病率) + 主题:(发生率) + 主题:(患病率) +主题:(罹患率) + 主题:(现患率) + 主题:(死亡率) +主题:(病死率) + 主题:(流行) + 主题:(负担)+ 主题:(现况调查) + 主题:(现况研究))
时间：1990-2017
Search Terms: (subject: (qingguangyan))* (subject: (fabinglv) + subject: (fashenglv) + subject: (huanbinglv)+ subject: (lihuanlv) + subject: (xianhuanlv) + subject: (siwanglv)+ subject: (bingsilv) + subject: (liuxing) + subject: (fudan)+ subject: (xiankuangdiaocha) + subject: (xiankuangyanjiu))
Date: 1990-2017

CBM-SinoMed
Access Date: 18 Aug 2017
Journal category: All journals
检索表达式：
(青光眼) AND (发病率 or 发生率 or 患病率 or 罹患率 or 现患率 or 死亡率 or 病死率 or 流行 or 负担 or 现况调查 or 现况研究)
时间：1990-2017
Search Terms: ((qingguangyan))* ((fabinglv) OR (fashenglv) OR (huanbinglv) OR (lihuanlv) OR (xianhuanlv) OR (siwanglv) OR (bingsilv) OR (liuxing) OR (fudan) OR (xiankuangdiaocha) OR (xiankuangyanjiu))
Date: 1990-2017

PubMed
Access Date: 18 Aug 2017
Search Terms:

((Glaucoma) AND (China OR Chinese OR Hongkong OR Macao OR Taiwan) AND (inciden* OR prevalen* OR morbidity OR mortality OR epidemiology)) AND ("1990/01/01"[Date - Publication] : "2017/08/18"[Date - Publication])

Embase (Ovid)
Access Date: 18 Aug 2017

#	Searches
**1**	exp glaucoma/ or Glaucoma.mp.
**2**	China.mp. or exp China/
**3**	exp Chinese/ or Chinese.mp.
**4**	Hong Kong.mp. or exp Hong Kong/
**5**	Macao.mp. or exp Macao/
**6**	Taiwan.mp. or exp Taiwan/
**7**	exp incidence/ or inciden*.mp.
**8**	exp prevalence/ or prevalen*.mp.
**9**	morbidity.mp. or morbidity/
**10**	mortality/ or Mortality.mp.
**11**	exp epidemiology/ or Epidemiology.mp.
**12**	2 or 3 or 4 or 5 or 6
**13**	7 or 8 or 9 or 10 or 11
**14**	1 and 12 and 13
**15**	limit 14 to yr="1990 -Current"

Medline (Ovid)
Access Date: 18 Aug 2017
Search Terms:

#	Searches
**1**	Glaucoma.mp. or exp Glaucoma/
**2**	China.mp. or exp China/
**3**	Chinese.mp.
**4**	Hong Kong.mp. or exp Hong Kong/
**5**	Macao.mp. or exp Macau/
**6**	Taiwan.mp. or exp Taiwan/

**7**	exp Incidence/ or inciden*.mp.
**8**	exp Prevalence/ or prevalen*.mp.
**9**	morbidity.mp. or exp Morbidity/
**10**	mortality.mp. or exp Mortality/
**11**	epidemiology.mp. or exp Epidemiology/
**12**	2 or 3 or 4 or 5 or 6
**13**	7 or 8 or 9 or 10 or 11
**14**	1 and 12 and 13
**15**	limit 14 to yr="1990 -Current"

Table S2. Meta-analysis of the prevalence of glaucoma for assessing heterogeneity between studies

To address the issue of heterogeneity across all eligible studies, the Cochran's Q and I^2 statistics were calculated. A p-value<0.05 indicates heterogeneity between studies in Q statistic, and I^2 represents the proportion of total variation that is due to heterogeneity rather than chance, where values of 25%, 50% and 75% representing low, moderate and high heterogeneity, respectively [72, 73]. As shown in Figures S1-S3, significant high heterogeneity was detected between studies that reported prevalence rates of POAG (I^2=92.5%, p<0.0001), PACG (I^2=88.2%, p<0.0001), and moderate heterogeneity existed between studies on secondary glaucoma (I^2=63.6%, p=0.0015).

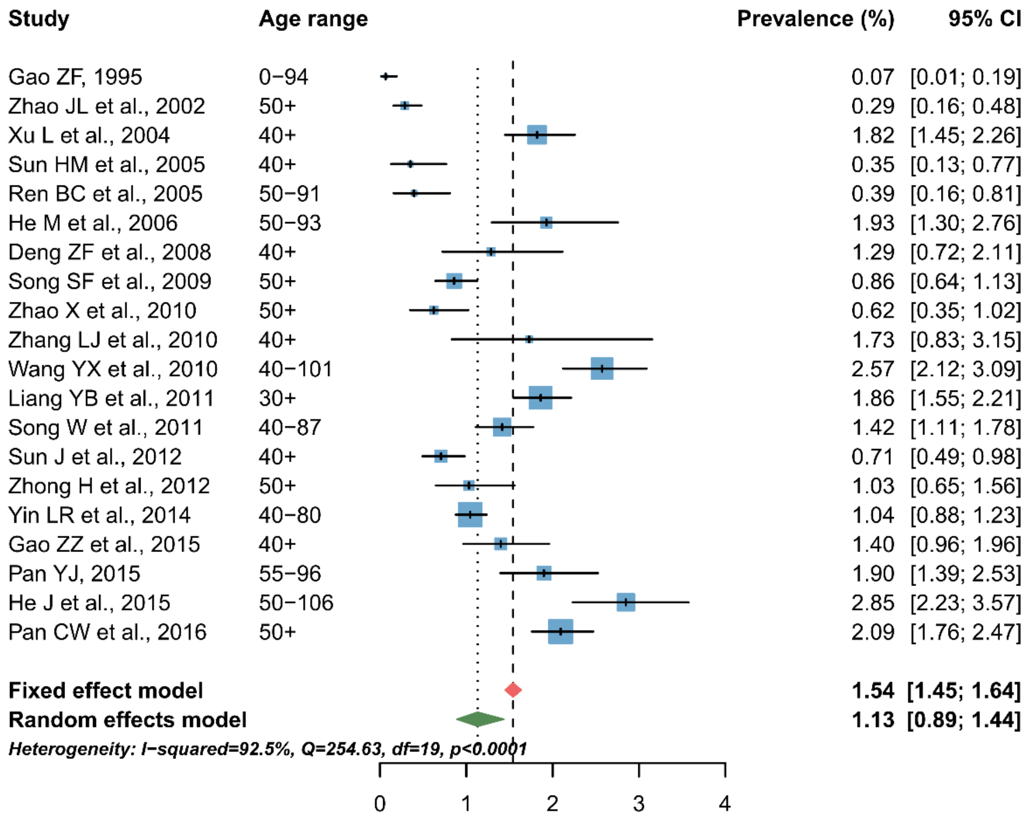

Figure S1. Meta-analysis of the prevalence of POAG (n=20)

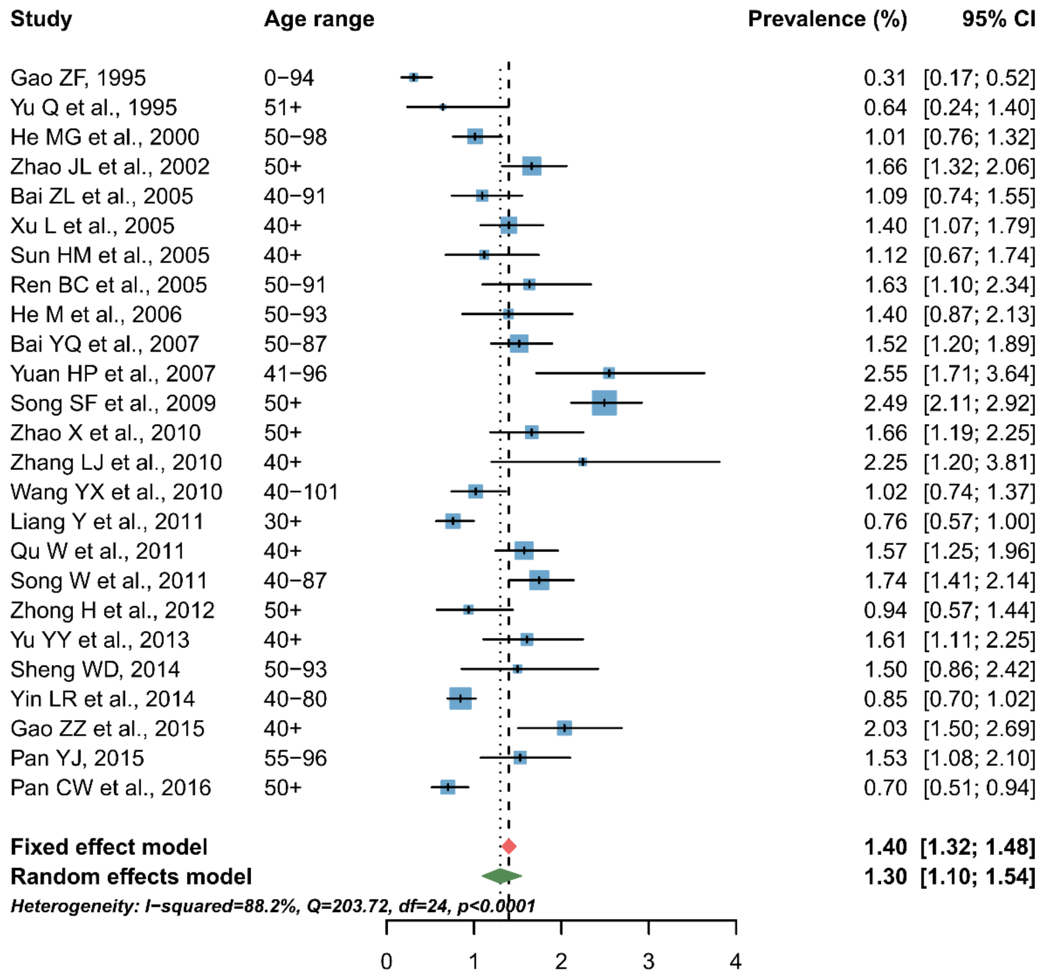

Figure S2. Meta-analysis of the prevalence of PACG (n=25)

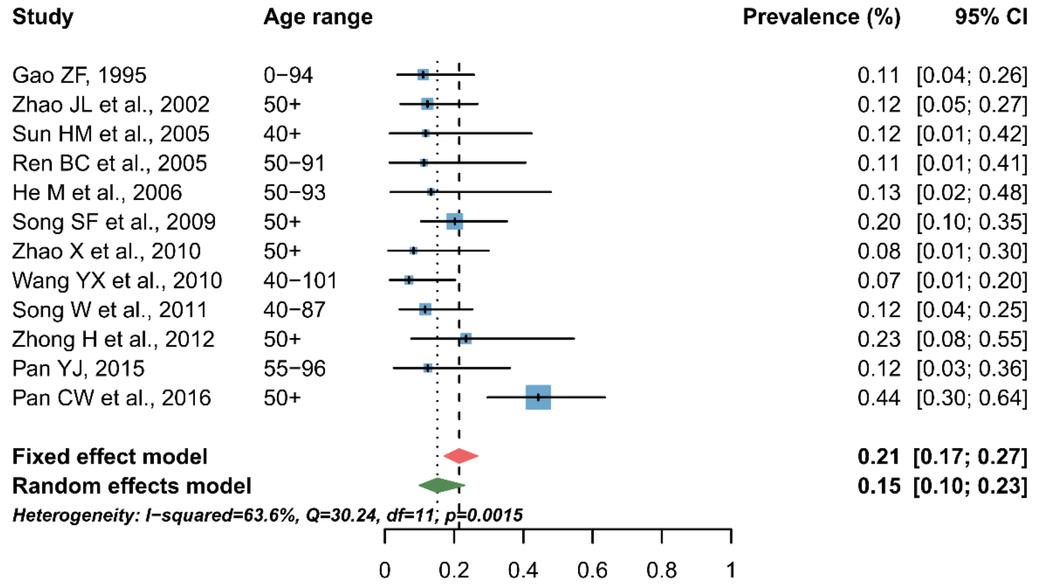

Figure S3. Meta-analysis of the prevalence of secondary glaucoma (n=12)

Table S3. Full list of the included studies on glaucoma prevalence in China (n=30)

Study ID	Reference
G-01	Zong-feng Gao. 高宗峰. An epidemiologic study of glaucoma in Tongcheng county, Anhui province (安徽省桐城县青光眼流行病学调查)[J]. Chinese Journal of Ophthalmology (中华眼科杂志). 1995(02):149-51.
G-02	Qiang Yu, Jing-jing Xu, Si-ping Zhu, et al. 于强, 许京京, 朱斯平, 柳青. An epidemiological survey of primary angle-closure glaucoma in Doumen county Guangdong (广东省斗门县原发性闭角型青光眼流行病学调查)[J]. Chinese Journal of Ophthalmology (中华眼科杂志). 1995(02):118-21.
G-03	Ming-guang He, Jing-jing Xu, Kai-li Wu, et al. 何明光, 许京京, 吴开力, 李绍珍. The prevalence of primary angle-closure glaucoma in elderly rural population (斗门县农村中老年人群原发闭角性青光眼流行病学调查)[J]. Acad J SUMS (中山医科大学学报). 2000(03):212-4.
G-04	Jia-liang Zhao, Rui-fang Sui, Li-jun Jia, et al. 赵家良, 睢瑞芳, 贾丽君, et al. Prevalence of glaucoma and normal intraocular pressure among adults aged 50 years or above in Shunyi county of Beijing (北京市顺义县 50 岁及以上人群中青光眼患病率和正常眼眼压的调查)[J]. Chin J Ophthalmol (中华眼科杂志). 2002(06):18-22.
G-05	Liang Xu, Jian-hua Chen, Jian-jun Li, et al. 徐亮, 陈建华, 李建军, et al. The prevalence and its screening methods of primary open angle glaucoma in defined population-based study of rural and urban in Beijing (北京农村及城市特定人群原发性开角型青光眼的患病率调查及其筛查方法评价)[J]. Chin J Ophthalmol (中华眼科杂志). 2004(11):9-15.
G-06	Lan-zhi Bai, Bai-chao Ren, Jian-gang Yang, et al. 白芝兰, 任百超, 杨建刚, 何媛, 陈莉, 孙乃学. Epidemiology of primary angle-closure glaucoma in a rural population in Shaanxi province of China (中国陕西省农村原发性闭角型青光眼流行病学调查)[J]. International journal of ophthalmology (国际眼科杂志). 2005;5(5):872-80.
G-07	Liang Xu, Li Zhang, Cui-ran Xia, et al. 徐亮, 张莉, 夏翠然, et al. The prevalence and its effective factors of primary angle-closure glaucoma in defined population of rural and urban in Beijing (北京农村及城市特定人群原发性闭角型青光眼的患病率及其影响因素)[J]. Chin J Ophthalmol (中华眼科杂志). 2005(01):12-8.
G-08	Hui-min Sun, Xiu-juan Zhang, Zhi-qing Li, et al. 孙慧敏, 张秀娟, 李志清, et al. Prevalence of glaucoma in Sangzi village, Ji county of Tianjin aged 40 years and above (天津市蓟县桑梓村 40 岁及以上人群中青光眼患病率调查)[J]. Chin J Pract Ophthalmol (中国实用眼科杂志). 2005(08):782-4.
G-09	Bai-chao Ren, Yuan He, Li Chen, et al. 任百超, 何媛, 陈莉, 杨建刚, 孙乃学. Epidemiology of glaucoma in a rural population in Shaanxi province (陕西省农村人群青光眼的流行病学调查)[J]. International journal of ophthalmology (国际眼科杂志). 2005(05):214-9.
G-10	Yong-quan Bai, Jing-lin Yi, Hui Xie, et al. 白永泉, 易敬林, 谢晖, et al. Epidemiological survey of primary angle-closure glaucoma in rural population aged 50 and elderly (吉安县农村 50 岁以上人群原发性闭角型青光眼流行病学调查)[J]. Medical Information section of operative surgery (医学信息(手术学分册)). 2007(09):774-7.

G-11	Hui-ping Yuan, Hong Yu, Zheng Xiao, et al. 原慧萍, 于泓, 肖铮, et al. The prevalence of primary angle-closure glaucoma and its causes in rural area of Shuangyang district in Changchun, Jilin province (吉林省长春市双阳区齐家乡原发性闭角青光眼的患病率调查及其影响因素)[J]. Chin J Ophthalmol (中华眼科杂志). 2007;43(9):775-8.
G-12	Zhi-feng Deng, Hong-juan Zhang. 邓志峰, 张洪娟. Epidemiology of primary open angle glaucoma in rural population in Heze (农村原发性开角型青光眼流行病学研究)[J]. Journal of Heze Medical College (菏泽医学专科学校学报). 2008(03):58-9.
G-13	Sheng-fang Song, Yong-ye Zhang, Xiang-ge He, et al. 宋胜仿, 张永烨, 贺翔鸽, et al. Prevalence of glaucoma among adults aged 50 years or above in Yongchuan district of Chongqing (重庆市永川地区 50 岁以上人群中青光眼患病率调查)[J]. Chin J Pract Ophthalmol (中国实用眼科杂志). 2009;27(2):168-72.
G-14	Xin Zhao, Yun-he He, Bi-qi Tian, et al. 赵欣, 郝云鹤, 田碧琪, et al. Glaucoma survey in population of 50 years old or more in the Xi Chang'an street community of Beijing (北京市西长安街社区 50 岁以上人群青光眼调查)[J]. Ophthalmol CHN (眼科). 2010(01):37-42.
G-15	Li-juan Zhang, Li Shan, Pan Fan, et al. 张丽娟, 单丽, 樊攀, 宋武莲, 原慧萍. Prevalence investigation on primary glaucoma in Kailu county, Inner Mongolia* (内蒙古开鲁县蒙古族原发性青光眼的患病率调查)[J]. Inner Mongolia Med J (内蒙古医学杂志). 2010(07):817-9.
G-16	Yang-yang Yu, Su-yun Wang, Shao-wei Wang. 于洋洋, 王素云, 王绍伟. Survey of prevalence of primary angle-closure glaucoma in rural population of Zhaozhou county (肇州县农村房角关闭疾病患病率调查)[J]. Chin J School Doctor (中国校医). 2013(08):599-601.
G-17	Wei-dong Sheng. 绳伟东. Epidemiological investigation on primary angle-closure glaucoma in middle-and-old aged people in Zhalantun city, Inner Mongolia* (内蒙古扎兰屯市中老年人群中原发性闭角型青光眼流行病学调查)[J]. China Prac Med (中国实用医药). 2014(24):261-3.
G-18	Lian-rong Yin, Hua Yang, Xin Li, et al. 尹连荣, 杨华, 李欣, 高健生. Primary discussion for education and screening on glaucoma in the western community in Beijing (京西社区青光眼宣教及筛查的初步探讨)[J]. Chinese Journal of Chinese Ophthalmology (中国中医眼科杂志). 2014(06):437-9.
G-19	Zhi-zhuo Gao, Tong Li, Yi-yuan Sun, et al. 高志卓, 李童, 孙艺源, et al. Epidemiological investigation of primary glaucoma in cold region of northern China (我国北方寒冷地区原发性青光眼流行病学调查分析)[J]. Chin J of Public Health Eng (中国卫生工程学). 2015(06):552-4.
G-20	Yu-jin Pan. 潘裕锦. Prevalence of primary angle-closure glaucoma: a over 55 years population based survey in Sijihuacheng community of Shenzhen (深圳市四季花城社区 55 岁以上人群原发性闭角型青光眼的流行病学调查)[D]. Jinan University (暨南大学), 2015.
G-21	He M, Foster PJ, Ge J, et al. Prevalence and clinical characteristics of glaucoma in adult Chinese: a population-based study in Liwan District, Guangzhou. Invest Ophthalmol Vis Sci 2006;47(7):2782-8.
G-22	Wang YX, Xu L, Yang H, Jonas JB. Prevalence of glaucoma in North China: the Beijing Eye Study. AM J OPHTHALMOL 2010;150(6):917-24.
G-23	Liang YB, Friedman DS, Zhou Q, et al. Prevalence of primary open angle glaucoma in a rural adult Chinese population: the Handan eye study. Invest Ophthalmol Vis Sci 2011;52(11):8250-7.

G-24	Liang Y, Friedman DS, Zhou Q, et al. Prevalence and characteristics of primary angle-closure diseases in a rural adult Chinese population: the Handan Eye Study. Invest Ophthalmol Vis Sci 2011;52(12):8672-9.
G-25	Qu W, Li Y, Song W, et al. Prevalence and risk factors for angle-closure disease in a rural Northeast China population: a population-based survey in Bin County, Harbin. ACTA OPHTHALMOL 2011;89(6):e515-20.
G-26	Song W, Shan L, Cheng F, et al. Prevalence of glaucoma in a rural northern China adult population: a population-based survey in kailu county, inner Mongolia. OPHTHALMOLOGY 2011;118(10):1982-8.
G-27	Sun J, Zhou X, Kang Y, et al. Prevalence and risk factors for primary open-angle glaucoma in a rural northeast China population: a population-based survey in Bin County, Harbin. Eye (Lond) 2012;26(2):283-91.
G-28	Zhong H, Li J, Li C, et al. The prevalence of glaucoma in adult rural Chinese populations of the Bai nationality in Dali: the Yunnan Minority Eye Study. Invest Ophthalmol Vis Sci 2012;53(6):3221-5.
G-29	He J, Zou H, Lee RK, et al. Prevalence and risk factors of primary open-angle glaucoma in a city of Eastern China: a population-based study in Pudong New District, Shanghai. BMC OPHTHALMOL 2015;15:134.
G-30	Pan CW, Zhao CH, Yu MB, et al. Prevalence, types and awareness of glaucoma in a multi-ethnic population in rural China: the Yunnan Minority Eye Study. Ophthalmic Physiol Opt 2016;36(6):664-70.

Note: The Chinese publication list employed the journals' official English names or abbreviations, English titles were obtained from journals or literature databases (CNKI, Wanfang and CBM). Where official English translation of journal names was not available, a pinyin title was adopted; where the English translation of titles was not available, the authors translated the titles, labelled with "" and marked as green.*

Table S4. Detailed characteristics of the included studies on glaucoma prevalence in China (n=30)

Study ID	Study	Province	Region	Setting	Gender	Survey Year	Anterior chamber angle/depth evaluation	IOP measurement	Optic disc evaluation	Visual field testing	Age range	Sample size	POAG	PACG	Secondary glaucoma
**G-01**	Gao ZF (1995)	Anhui	East China	Rural	Both	1987	Yes, all	Yes, all	Yes, suspects	Yes, suspects	0-94	4531	3	14	5
**G-02**	Yu Q et al. (1995)	Guangdong	South Central China	Rural	Mixed	1990	Yes, all	Yes, all	Yes, suspects	Yes, suspects	51+	932	-	6	-
**G-03**	He MG et al. (2000)	Guangdong	South Central China	Rural	Both	1997	Yes, all	Yes, all	Yes, all	Yes, suspects	50-98	5342	-	54	-
**G-04**	Zhao JL et al. (2002)	Beijing	North China	Mixed	Both	1996	Yes, all	Yes, all	Yes, all	Yes, suspects	50+	4880	14	81	6
**G-05**	Xu L et al. (2004)	Beijing	North China	Both	Both	2001	Yes, suspects	Yes, all	Yes, all	Yes, all	40+	4451	81	-	-
**G-06**	Bai ZL et al. (2005)	Shaanxi	Northwest China	Rural	Both	2003	Yes, all	Yes, all	Yes, all	Yes, suspects	40-91	2835	-	31	-
**G-07**	Xu L et al. (2005)	Beijing	North China	Both	Both	2001	Yes, suspects	Yes, all	Yes, all	Yes, all	40+	4431	-	62	-
**G-08**	Sun HM et al. (2005)	Tianjin	North China	Rural	Both	2003	Yes, all	Yes, all	Yes, all	Yes, suspects	40+	1701	6	19	2
**G-09**	Ren BC et al. (2005)	Shaanxi	Northwest China	Rural	Both	2003	Yes, all	Yes, all	Yes, all	Yes, suspects	50-91	1775	7	29	2
**G-10**	Bai YQ et al. (2007)	Jiangxi	East China	Rural	Both	2006	Yes, all	Yes, suspects	Yes, all	Yes, suspects	50-87	5013	-	76	-
**G-11**	Yuan HP et al. (2007)	Jilin	Northeast China	Rural	Both	2004	Yes, all	Yes, all	Yes, all	Yes, suspects	41-96	1139	-	29	-
**G-12**	Deng ZF et al. (2008)	Shandong	East China	Rural	Both	2005*	Yes, all	Yes, all	Yes, all	Yes, suspects	40+	1166	15	-	-
**G-13**	Song SF et al. (2009)	Chongqing	Southwest China	Mixed	Both	2005	Yes, all	Yes, all	Yes, all	Yes, suspects	50+	5938	51	148	12
**G-14**	Zhao X et al. (2010)	Beijing	North China	Urban	Both	2006	Yes, all	Yes, all	Yes, all	Yes, suspects	50+	2410	15	40	2

**G-15**	Zhang LJ et al. (2010)	Inner Mongolia	North China	Rural	Both	2009	Yes, all	Yes, all	Yes, all	Yes, suspects	40+	579	10	13	-
**G-16**	Yu YY et al. (2013)	Heilongjiang	Northeast China	Rural	Both	2010*	Yes, all	Yes, all	Yes, all	Yes, suspects	40+	2056	-	33	-
**G-17**	Sheng WD (2014)	Inner Mongolia	North China	Mixed	Both	2013	Yes, all	Yes, all	Yes, all	Yes, suspects	50-93	1068	-	16	-
**G-18**	Yin LR et al. (2014)	Beijing	North China	Urban	Mixed	2010	Yes, all	Yes, all	Yes, all	Yes, all	40-80	13016	136	110	-
**G-19**	Gao ZZ et al. (2015)	Jilin	Northeast China	Urban	Both	2011	Yes, all	Yes, all	Yes, all	Yes, suspects	40+	2359	33	48	-
**G-20**	Pan YJ (2015)	Guangdong	South Central China	Urban	Both	2014	Yes, all	Yes, all	Yes, all	Yes, suspects	55-96	2422	46	37	3
**G-21**	He M et al. (2006)	Guangdong	South Central China	Urban	Both	2003	Yes, all	Yes, all	Yes, all	Yes, suspects	50-93	1504	29	21	2
**G-22**	Wang YX et al. (2010)	Beijing	North China	Both	Both	2001	Yes, all	Yes, all	Yes, all	Yes, all	40-101	4315	111	44	3
**G-23**	Liang YB et al. (2011)	Hebei	North China	Rural	Both	2007	Yes, all	Yes, all	Yes, all	Yes, suspects	30+	6716	125	-	-
**G-24**	Liang Y et al. (2011)	Hebei	North China	Rural	Both	2007	Yes, all	Yes, all	Yes, all	Yes, suspects	30+	6716	-	51	-
**G-25**	Qu W et al. (2011)	Heilongjiang	Northeast China	Rural	Both	2007	Yes, all	Yes, all	Yes, all	Yes, suspects	40+	4956	-	78	-
**G-26**	Song W et al. (2011)	Inner Mongolia	North China	Rural	Both	2009	Yes, all	Yes, all	Yes, all	Yes, suspects	40-87	5158	73	90	6
**G-27**	Sun J et al. (2012)	Heilongjiang	Northeast China	Rural	Both	2007	Yes, all	Yes, all	Yes, all	Yes, suspects	40+	4956	35	-	-
**G-28**	Zhong H et al. (2012)	Yunnan	Southwest China	Rural	Both	2010	Yes, all	Yes, all	Yes, all	Yes, suspects	50+	2133	22	20	5
**G-29**	He J et al. (2015)	Shanghai	East China	Urban	Both	2011	Yes, all	Yes, all	Yes, all	Yes, suspects	50-106	2528	72	-	-
**G-30**	Pan CW et al. (2016)	Yunnan	Southwest China	Rural	Both	2010	Yes, all	Yes, all	Yes, all	Yes, suspects	50+	6546	137	46	29

Note: "-" represents unavailable data; "" indicates studies whose survey year was imputed.*

Table S5. Estimate and projection of gender-specific number of people with POAG and PACG in China from 1990 to 2050, by age group (million, 95% CI)

1990						
Age	POAG			PACG		
	Male	Female	Overall	Male	Female	Overall
45-49 years	0.19 (0.12-0.29)	0.12 (0.08-0.19)	0.31 (0.20-0.48)	0.12 (0.10-0.15)	0.21 (0.17-0.26)	0.33 (0.27-0.41)
50-54 years	0.21 (0.14-0.32)	0.14 (0.09-0.21)	0.35 (0.23-0.53)	0.15 (0.12-0.18)	0.25 (0.21-0.29)	0.40 (0.33-0.47)
55-59 years	0.22 (0.15-0.34)	0.15 (0.10-0.23)	0.38 (0.25-0.57)	0.17 (0.14-0.20)	0.29 (0.25-0.34)	0.46 (0.39-0.54)
60-64 years	0.21 (0.14-0.32)	0.15 (0.10-0.22)	0.36 (0.23-0.54)	0.17 (0.14-0.20)	0.30 (0.26-0.35)	0.47 (0.40-0.55)
65-69 years	0.18 (0.12-0.28)	0.14 (0.09-0.21)	0.32 (0.21-0.48)	0.16 (0.13-0.19)	0.30 (0.26-0.35)	0.46 (0.39-0.53)
70-74 years	0.16 (0.11-0.25)	0.13 (0.08-0.20)	0.29 (0.19-0.44)	0.15 (0.12-0.18)	0.30 (0.26-0.36)	0.45 (0.38-0.54)
75-79 years	0.10 (0.07-0.16)	0.10 (0.06-0.15)	0.20 (0.13-0.31)	0.10 (0.08-0.13)	0.25 (0.21-0.30)	0.35 (0.29-0.42)
80–84 years	0.05 (0.03-0.07)	0.06 (0.04-0.09)	0.11 (0.07-0.17)	0.05 (0.04-0.06)	0.16 (0.13-0.20)	0.21 (0.17-0.26)
85-89 years	0.02 (0.01-0.03)	0.03 (0.02-0.04)	0.04 (0.03-0.07)	0.02 (0.01-0.02)	0.07 (0.06-0.09)	0.09 (0.07-0.12)
Total (45-89 years)	1.35 (0.88-2.05)	1.01 (0.66-1.55)	2.35 (1.54-3.60)	1.09 (0.90-1.32)	2.13 (1.80-2.53)	3.22 (2.70-3.84)
2000						
Age	POAG			PACG		
	Male	Female	Overall	Male	Female	Overall
45-49 years	0.32 (0.21-0.50)	0.23 (0.15-0.35)	0.55 (0.36-0.85)	0.21 (0.17-0.26)	0.38 (0.31-0.46)	0.59 (0.48-0.73)
50-54 years	0.27 (0.18-0.42)	0.19 (0.13-0.29)	0.46 (0.30-0.71)	0.19 (0.16-0.23)	0.35 (0.29-0.41)	0.54 (0.45-0.64)
55-59 years	0.25 (0.16-0.38)	0.17 (0.11-0.26)	0.42 (0.27-0.64)	0.19 (0.16-0.22)	0.33 (0.28-0.38)	0.51 (0.44-0.60)
60-64 years	0.27 (0.18-0.40)	0.18 (0.12-0.27)	0.45 (0.29-0.68)	0.22 (0.18-0.25)	0.37 (0.32-0.43)	0.59 (0.50-0.68)
65-69 years	0.26 (0.17-0.39)	0.19 (0.12-0.28)	0.44 (0.29-0.67)	0.22 (0.19-0.27)	0.41 (0.35-0.47)	0.63 (0.54-0.74)
70-74 years	0.21 (0.14-0.31)	0.16 (0.10-0.25)	0.37 (0.24-0.56)	0.19 (0.16-0.23)	0.38 (0.32-0.44)	0.57 (0.48-0.67)
75-79 years	0.14 (0.09-0.21)	0.13 (0.08-0.19)	0.27 (0.17-0.41)	0.14 (0.11-0.17)	0.32 (0.26-0.38)	0.45 (0.38-0.55)
80–84 years	0.09 (0.06-0.13)	0.09 (0.06-0.14)	0.18 (0.11-0.27)	0.09 (0.07-0.12)	0.24 (0.19-0.29)	0.33 (0.27-0.41)
85-89 years	0.03 (0.02-0.05)	0.04 (0.03-0.07)	0.08 (0.05-0.12)	0.04 (0.03-0.05)	0.12 (0.10-0.15)	0.16 (0.13-0.20)
Total (45-89 years)	1.84 (1.20-2.80)	1.37 (0.89-2.11)	3.21 (2.09-4.91)	1.49 (1.23-1.80)	2.88 (2.43-3.43)	4.37 (3.66-5.23)
2010						
Age	POAG			PACG		
	Male	Female	Overall	Male	Female	Overall
45-49 years	0.38	0.27	0.65	0.25	0.45	0.70

	(0.25-0.59)	(0.17-0.41)	(0.42-1.00)	(0.20-0.31)	(0.37-0.55)	(0.57-0.86)
50-54 years	0.36 (0.24-0.56)	0.26 (0.17-0.39)	0.62 (0.41-0.95)	0.26 (0.21-0.31)	0.46 (0.39-0.55)	0.72 (0.60-0.86)
55-59 years	0.44 (0.29-0.66)	0.31 (0.20-0.47)	0.75 (0.49-1.13)	0.33 (0.28-0.39)	0.60 (0.51-0.70)	0.93 (0.79-1.09)
60-64 years	0.35 (0.23-0.54)	0.26 (0.17-0.39)	0.61 (0.40-0.93)	0.29 (0.24-0.34)	0.53 (0.46-0.61)	0.81 (0.70-0.95)
65-69 years	0.30 (0.20-0.46)	0.21 (0.14-0.33)	0.51 (0.34-0.78)	0.26 (0.22-0.31)	0.47 (0.41-0.54)	0.73 (0.63-0.85)
70-74 years	0.28 (0.18-0.43)	0.21 (0.14-0.32)	0.49 (0.32-0.75)	0.26 (0.22-0.31)	0.49 (0.42-0.57)	0.75 (0.63-0.89)
75-79 years	0.22 (0.14-0.33)	0.18 (0.12-0.28)	0.40 (0.26-0.61)	0.22 (0.18-0.27)	0.46 (0.38-0.55)	0.67 (0.56-0.81)
80–84 years	0.13 (0.08-0.19)	0.12 (0.08-0.19)	0.25 (0.16-0.38)	0.13 (0.11-0.17)	0.32 (0.26-0.40)	0.46 (0.37-0.57)
85-89 years	0.06 (0.03-0.09)	0.06 (0.04-0.10)	0.12 (0.07-0.19)	0.06 (0.05-0.08)	0.18 (0.14-0.22)	0.24 (0.19-0.31)
Total (45-89 years)	2.51 (1.64-3.84)	1.88 (1.22-2.88)	4.39 (2.86-6.72)	2.05 (1.70-2.48)	3.95 (3.33-4.69)	6.01 (5.03-7.18)
**2015**						
**Age**	**POAG**			**PACG**		
	**Male**	**Female**	**Overall**	**Male**	**Female**	**Overall**
45-49 years	0.46 (0.30-0.71)	0.33 (0.21-0.51)	0.79 (0.51-1.22)	0.30 (0.24-0.38)	0.55 (0.45-0.68)	0.86 (0.70-1.05)
50-54 years	0.45 (0.29-0.68)	0.32 (0.21-0.48)	0.76 (0.50-1.16)	0.31 (0.26-0.38)	0.57 (0.48-0.67)	0.88 (0.74-1.05)
55-59 years	0.42 (0.28-0.64)	0.30 (0.20-0.46)	0.72 (0.47-1.10)	0.32 (0.27-0.38)	0.58 (0.50-0.67)	0.90 (0.76-1.05)
60-64 years	0.49 (0.32-0.75)	0.36 (0.23-0.54)	0.85 (0.56-1.29)	0.40 (0.34-0.47)	0.73 (0.64-0.85)	1.13 (0.97-1.32)
65-69 years	0.38 (0.25-0.58)	0.28 (0.19-0.43)	0.66 (0.44-1.01)	0.33 (0.28-0.39)	0.63 (0.54-0.72)	0.96 (0.82-1.11)
70-74 years	0.30 (0.20-0.46)	0.22 (0.15-0.34)	0.52 (0.34-0.80)	0.28 (0.23-0.33)	0.53 (0.45-0.62)	0.80 (0.68-0.95)
75-79 years	0.25 (0.16-0.38)	0.20 (0.13-0.31)	0.44 (0.29-0.68)	0.24 (0.20-0.30)	0.50 (0.41-0.60)	0.74 (0.61-0.90)
80–84 years	0.16 (0.10-0.25)	0.15 (0.10-0.23)	0.31 (0.20-0.48)	0.17 (0.14-0.21)	0.40 (0.32-0.49)	0.57 (0.46-0.70)
85-89 years	0.07 (0.05-0.11)	0.08 (0.05-0.12)	0.15 (0.10-0.24)	0.08 (0.06-0.11)	0.22 (0.17-0.28)	0.31 (0.24-0.39)
Total (45-89 years)	2.98 (1.95-4.55)	2.24 (1.45-3.43)	5.22 (3.40-7.98)	2.44 (2.01-2.95)	4.70 (3.96-5.58)	7.14 (5.97-8.53)
**2020**						
**Age**	**POAG**			**PACG**		
	**Male**	**Female**	**Overall**	**Male**	**Female**	**Overall**
45-49 years	0.44 (0.29-0.68)	0.31 (0.20-0.48)	0.76 (0.49-1.17)	0.29 (0.23-0.36)	0.52 (0.43-0.64)	0.81 (0.66-1.00)
50-54 years	0.54 (0.35-0.83)	0.39 (0.25-0.60)	0.93 (0.61-1.42)	0.38 (0.31-0.46)	0.70 (0.59-0.83)	1.08 (0.90-1.29)
55-59 years	0.52 (0.34-0.79)	0.37 (0.24-0.56)	0.89 (0.58-1.35)	0.39 (0.33-0.46)	0.71 (0.61-0.83)	1.10 (0.94-1.29)
60-64 years	0.48 (0.32-0.73)	0.35 (0.23-0.53)	0.82 (0.54-1.25)	0.39 (0.33-0.46)	0.71 (0.62-0.82)	1.10 (0.94-1.28)
65-69 years	0.53	0.40	0.93	0.46	0.88	1.34

	(0.35-0.81)	(0.26-0.61)	(0.61-1.42)	(0.39-0.55)	(0.76-1.02)	(1.15-1.57)
70-74 years	0.38 (0.25-0.59)	0.30 (0.20-0.46)	0.69 (0.45-1.05)	0.36 (0.30-0.43)	0.71 (0.60-0.83)	1.07 (0.90-1.26)
75-79 years	0.27 (0.17-0.41)	0.22 (0.14-0.34)	0.49 (0.31-0.75)	0.27 (0.22-0.33)	0.55 (0.45-0.66)	0.81 (0.67-0.98)
80–84 years	0.19 (0.12-0.29)	0.17 (0.11-0.26)	0.35 (0.23-0.55)	0.20 (0.16-0.25)	0.45 (0.36-0.55)	0.65 (0.52-0.80)
85-89 years	0.10 (0.06-0.15)	0.10 (0.06-0.16)	0.20 (0.13-0.31)	0.11 (0.09-0.14)	0.28 (0.22-0.36)	0.40 (0.31-0.50)
Total (45-89 years)	3.45 (2.26-5.28)	2.60 (1.69-3.99)	6.06 (3.95-9.27)	2.84 (2.35-3.44)	5.51 (4.65-6.54)	8.36 (7.00-9.98)
**2030**						
**Age**	**POAG**			**PACG**		
	**Male**	**Female**	**Overall**	**Male**	**Female**	**Overall**
45-49 years	0.37 (0.24-0.57)	0.26 (0.17-0.40)	0.62 (0.40-0.96)	0.24 (0.19-0.30)	0.43 (0.35-0.53)	0.67 (0.55-0.83)
50-54 years	0.42 (0.27-0.64)	0.29 (0.19-0.45)	0.71 (0.46-1.08)	0.29 (0.24-0.35)	0.53 (0.44-0.63)	0.82 (0.68-0.98)
55-59 years	0.61 (0.40-0.93)	0.43 (0.28-0.66)	1.04 (0.68-1.59)	0.46 (0.39-0.55)	0.83 (0.72-0.97)	1.29 (1.10-1.52)
60-64 years	0.72 (0.48-1.10)	0.53 (0.35-0.81)	1.25 (0.82-1.90)	0.58 (0.49-0.69)	1.09 (0.94-1.26)	1.68 (1.44-1.95)
65-69 years	0.65 (0.43-0.99)	0.48 (0.32-0.74)	1.14 (0.75-1.73)	0.57 (0.48-0.67)	1.06 (0.92-1.23)	1.63 (1.40-1.90)
70-74 years	0.55 (0.36-0.83)	0.42 (0.27-0.65)	0.97 (0.63-1.48)	0.51 (0.42-0.61)	0.99 (0.84-1.16)	1.50 (1.27-1.77)
75-79 years	0.52 (0.34-0.79)	0.43 (0.28-0.66)	0.95 (0.61-1.46)	0.51 (0.42-0.63)	1.08 (0.90-1.30)	1.59 (1.32-1.93)
80–84 years	0.29 (0.18-0.45)	0.27 (0.17-0.42)	0.55 (0.36-0.86)	0.31 (0.24-0.38)	0.71 (0.58-0.87)	1.02 (0.82-1.26)
85-89 years	0.14 (0.09-0.22)	0.14 (0.09-0.22)	0.28 (0.18-0.44)	0.16 (0.12-0.20)	0.39 (0.31-0.50)	0.55 (0.43-0.70)
Total (45-89 years)	4.26 (2.78-6.51)	3.25 (2.12-4.99)	7.51 (4.90-11.5)	3.63 (3.00-4.39)	7.12 (6.00-8.45)	10.75 (9.00-12.84)
**2040**						
**Age**	**POAG**			**PACG**		
	**Male**	**Female**	**Overall**	**Male**	**Female**	**Overall**
45-49 years	0.40 (0.26-0.62)	0.27 (0.17-0.41)	0.66 (0.43-1.03)	0.26 (0.21-0.32)	0.45 (0.36-0.54)	0.71 (0.57-0.87)
50-54 years	0.57 (0.37-0.87)	0.39 (0.25-0.60)	0.96 (0.63-1.47)	0.40 (0.33-0.48)	0.70 (0.59-0.83)	1.10 (0.92-1.32)
55-59 years	0.51 (0.33-0.77)	0.36 (0.23-0.55)	0.87 (0.57-1.32)	0.38 (0.32-0.46)	0.69 (0.59-0.80)	1.07 (0.91-1.26)
60-64 years	0.56 (0.37-0.85)	0.40 (0.26-0.61)	0.96 (0.63-1.46)	0.45 (0.38-0.54)	0.83 (0.72-0.95)	1.28 (1.10-1.49)
65-69 years	0.79 (0.52-1.20)	0.58 (0.38-0.88)	1.36 (0.90-2.07)	0.68 (0.58-0.81)	1.27 (1.09-1.47)	1.95 (1.67-2.28)
70-74 years	0.86 (0.57-1.32)	0.66 (0.43-1.01)	1.53 (1.00-2.33)	0.80 (0.67-0.96)	1.56 (1.33-1.83)	2.36 (2.00-2.79)
75-79 years	0.68 (0.44-1.04)	0.55 (0.35-0.84)	1.23 (0.79-1.89)	0.67 (0.55-0.83)	1.37 (1.14-1.65)	2.05 (1.69-2.47)
80–84 years	0.46 (0.29-0.71)	0.40 (0.26-0.63)	0.86 (0.55-1.33)	0.49 (0.39-0.61)	1.07 (0.87-1.32)	1.56 (1.26-1.93)
85-89 years	0.31	0.31	0.62	0.35	0.87	1.22

	(0.20-0.48)	(0.19-0.49)	(0.39-0.97)	(0.27-0.45)	(0.69-1.10)	(0.96-1.56)
Total (45-89 years)	5.14 (3.35-7.85)	3.91 (2.54-6.01)	9.04 (5.89-13.86)	4.49 (3.70-5.46)	8.81 (7.38-10.50)	13.3 (11.08-15.97)
**2050**						
**Age**	**POAG**			**PACG**		
	**Male**	**Female**	**Overall**	**Male**	**Female**	**Overall**
45-49 years	0.29 (0.19-0.44)	0.18 (0.12-0.28)	0.47 (0.30-0.72)	0.19 (0.15-0.23)	0.31 (0.25-0.37)	0.49 (0.40-0.61)
50-54 years	0.35 (0.23-0.54)	0.23 (0.15-0.35)	0.58 (0.38-0.89)	0.25 (0.20-0.30)	0.41 (0.35-0.49)	0.66 (0.55-0.79)
55-59 years	0.56 (0.37-0.85)	0.37 (0.24-0.56)	0.93 (0.61-1.41)	0.42 (0.35-0.50)	0.71 (0.61-0.83)	1.13 (0.96-1.33)
60-64 years	0.78 (0.51-1.18)	0.54 (0.35-0.82)	1.32 (0.86-2.00)	0.63 (0.53-0.74)	1.11 (0.96-1.28)	1.74 (1.49-2.02)
65-69 years	0.67 (0.44-1.02)	0.48 (0.31-0.73)	1.15 (0.76-1.75)	0.58 (0.49-0.69)	1.06 (0.91-1.23)	1.64 (1.41-1.92)
70-74 years	0.70 (0.46-1.07)	0.51 (0.33-0.79)	1.21 (0.79-1.85)	0.65 (0.54-0.78)	1.21 (1.03-1.42)	1.86 (1.57-2.20)
75-79 years	0.88 (0.57-1.34)	0.68 (0.44-1.04)	1.55 (1.01-2.39)	0.87 (0.71-1.07)	1.70 (1.41-2.04)	2.57 (2.13-3.10)
80–84 years	0.80 (0.51-1.24)	0.67 (0.43-1.05)	1.47 (0.94-2.29)	0.85 (0.68-1.07)	1.80 (1.46-2.21)	2.65 (2.13-3.28)
85-89 years	0.47 (0.30-0.73)	0.43 (0.27-0.68)	0.90 (0.57-1.41)	0.53 (0.41-0.69)	1.22 (0.96-1.55)	1.76 (1.37-2.24)
Total (45-89 years)	5.49 (3.57-8.41)	4.10 (2.65-6.31)	9.59 (6.23-14.72)	4.97 (4.07-6.07)	9.52 (7.94-11.41)	14.49 (12.01-17.48)

REFERENCES - SUPPLEMENTARY ONLINE MATERIAL

72. Higgins, J. and S.G. Thompson, *Quantifying heterogeneity in a meta-analysis.* Statistics in medicine, 2002. **21**(11): p. 1539-1558.
73. Higgins, J.P., et al., *Measuring inconsistency in meta-analyses.* BMJ, 2003. **327**(7414): p. 557-560.
